# Bivalent promoter hypermethylation in cancer is linked to the H327me3/H3K4me3 ratio in embryonic stem cells

**DOI:** 10.1186/s12915-020-0752-3

**Published:** 2020-03-04

**Authors:** Donnchadh S. Dunican, Heidi K. Mjoseng, Leanne Duthie, Ilya M. Flyamer, Wendy A. Bickmore, Richard R. Meehan

**Affiliations:** MRC Human Genetics Unit, MRC IGMM, University of Edinburgh, Western General Hospital, Crewe Road, Edinburgh, EH4 2XU Scotland

**Keywords:** DNA methylation, Polycomb, Bivalent, Epigenetic, Chromatin, Development, Cancer, Stem cells, Trithorax

## Abstract

**Background:**

Thousands of mammalian promoters are defined by co-enrichment of the histone tail modifications H3K27me3 (repressive) and H3K4me3 (activating) and are thus termed bivalent. It was previously observed that bivalent genes in human ES cells (hESC) are frequent targets for hypermethylation in human cancers, and depletion of DNA methylation in mouse embryonic stem cells has a marked impact on H3K27me3 distribution at bivalent promoters. However, only a fraction of bivalent genes in stem cells are targets of hypermethylation in cancer, and it is currently unclear whether all bivalent promoters are equally sensitive to DNA hypomethylation and whether H3K4me3 levels play a role in the interplay between DNA methylation and H3K27me3.

**Results:**

We report the sub-classification of bivalent promoters into two groups—promoters with a high H3K27me3:H3K4me3 (hiBiv) ratio or promoters with a low H3K27me3:H3K4me3 ratio (loBiv). HiBiv are enriched in canonical Polycomb components, show a higher degree of local intrachromosomal contacts and are highly sensitive to DNA hypomethylation in terms of H3K27me3 depletion from broad Polycomb domains. In contrast, loBiv promoters are enriched in non-canonical Polycomb components, show lower intrachromosomal contacts and are less sensitive to DNA hypomethylation at the same genomic resolution. Multiple systems reveal that hiBiv promoters are more depleted of Polycomb complexes than loBiv promoters following a reduction in DNA methylation, and we demonstrate that H3K27me3 re-accumulates at promoters when DNA methylation is restored. In human cancer, we show that hiBiv promoters lose H3K27me3 and are more susceptible to DNA hypermethylation than loBiv promoters.

**Conclusion:**

We conclude that bivalency as a general term to describe mammalian promoters is an over-simplification and our sub-classification has revealed novel insights into the interplay between the largely antagonistic presence of DNA methylation and Polycomb systems at bivalent promoters. This approach redefines molecular pathologies underlying disease in which global DNA methylation is aberrant or where Polycomb mutations are present.

**Supplementary information:**

**Supplementary information** accompanies this paper at 10.1186/s12915-020-0752-3.

## Background

In the mammalian genome, DNA methylation at the 5′ position of cytosine (5meC) is required for normal embryonic development [1]. In addition, DNA methylation has been shown to play important roles in repetitive element silencing, X-inactivation and genomic imprinting [2–4]. Methylation is abundant at interspersed CpG dinucleotides (CG) and is depleted from CpG island (CGI) regions of high CG content which are often (~ 70%) associated with gene promoters [5]. Additionally, the presence of 5meC at promoter regions is generally correlated with transcriptional inactivity [5].

Transcriptional repression and developmental gene regulation are also controlled by the action of two major classes of polycomb repressive complexes (PRCs). PRC2 mediates the methylation of lysine 27 on histone H3 (H3K27me3), via the SET-domain containing methyltransferase activity of EZH2, which is an epigenetic mark associated with transcriptional silencing [6]. In parallel, PRC1 ubiquitylates lysine 119 of histone H2A (uH2A), utilising the E3 ligase activity of RING1B. PRC1 is associated with chromatin compaction—although the presence of catalytically inactive RING1B is sufficient for the compacted state [7]. Biochemical studies of PRC proteins have revealed several complexes with distinct compositions and functions with the potential for significant combinatorial diversity in each class of complexes. In particular, canonical PRC1 complexes contain a Cbx subunit and either PCGF2 or PCGF4 [8], while non-canonical PRC1 variant complexes may contain other components including RYBP or KDM2B instead of other PCGF proteins [9]. Deposition of PRC histone marks can arise by either PRC2-dependent recruitment of PRC1, or vice versa [10, 11], and the combination of PRC1 and PRC2 is thought to be essential for developmental gene regulation and thus lineage specification. Moreover, their importance is exemplified by the human diseases involving PRC alterations: Weaver syndrome, Ataxia telangiectasia and autism spectrum disorders [12–14]. PRC2 (*Ezh2*^*−/−*^*, Eed*^*−/−*^ or *Suz12*^*−/−*^) mouse knockouts are lethal at postimplantation while PRC1 (*Ring1b*^*−/−*^ or *Kdm2b*^*−/−*^) mice die subsequent to gastrulation [15–19].

An important aspect of epigenetic repression is that 5meC and H3K27me3 are rarely found coincident at the same location in the genome [20]. For example, developmentally regulated homeobox gene clusters are rich in PRC marks, but relatively depleted of 5meC [21]. In contrast, interspersed repetitive sequences are enriched for high levels of 5meC and relatively low levels of PRC marks. Thus, it is possible that one role of 5meC is to constrain PRC activity to high CG content regions of mammalian genomes and inhibit its deposition at lower CG content regions [22]. Alternatively, perhaps the role of PRC is to prevent the spread of 5meC into gene promoters which may have long-term negative effects on transcriptional output.

Bivalent promoters are enriched for both repressive PRC components and the activation-associated mark trimethylation of lysine 4 of histone H3 (H3K4me3) [23, 24]. Biochemical studies have shown that individual H3 histones on given nucleosomes can be asymmetrically marked with H3K27me3 and H3K4me3 [25]. The Trithorax Group (TrX) member MLL2 (KMT2D; WBP7) deposits H3K4me3 at promoters [26]. In the absence of H3K27me3, H3K4me3 marked promoters are largely transcriptionally active and contain high levels of RNA polymerase II (RPII) phosphorylated on serine 2 (S2P-CTD) of the C-terminal heptapeptide-repeat domain (CTD) which is associated with transcriptional elongation [27]. H3K27me3 and H3K4me3 co-occupied promoters are termed ‘poised’, in that resolution of either mark is correlated with transcriptional output: forced transcription can remove H3K27me3 while loss of H3K4me3 reduces transcription [28–31]. Importantly, bivalent promoters are 5meC depleted, in part due to the presence of H3K27me3 [32]. Interestingly, loss of MLL2 leads to accumulation of H3K27me3 at previously bivalent promoters; therefore, one role of MLL2-dependent H3K4me3 is to constrain over-accumulation of H3K27me3 at bivalent promoters which may affect the ‘poised’ equilibrium between these opposing bivalent chromatin demarcations [33].

Loss of 5meC from repetitive DNA in *Dnmt1−/−* knockout somatic cells leads to sequestration of Ezh2-dependent H3K27me3 from PRC target genes to intergenic regions [21, 34]. Efforts have been made to investigate whether this finding is restricted to differentiated cell types [20, 35, 36]; however, these studies generally utilised a broad definition of bivalent promoters as a single class. Moreover, it is unknown if PRC compartmentalisation is affected in systems where 5meC is acutely depleted and subsequently restored. An interesting association between bivalency and 5meC was the finding that many bivalent genes in human ES cells (hESC) are frequent targets for hypermethylation in human cancers [37, 38]. Moreover, aberrant DNA hypermethylation is observed at H3K27me3 enriched regions in microcephalic dwarfism patient-derived fibroblasts harbouring the DNMT3A^W330R/+^ genotype within the PWWP domain [39]. It remains incompletely understood whether all broadly categorised bivalent genes respond to global hypomethylation in pluripotent embryonic stem (ES) cells, and if these represent primary targets for hypermethylation in transformed cancer cells.

Here, we characterise mESC lacking 5meC and show severely disrupted H3K27me3 targeting. Clustering approaches reveal two distinct classes of bivalent gene promoters which differ in hypomethylation response, expression and three-dimensional chromatin organisation. We extend these findings to other aberrant 5meC systems and show that PRC mis-targeting is reversible. Finally, we demonstrate that a distinct group of hESC bivalent promoters are preferentially targeted for promoter hypermethylation in human cancer.

## Results

### Hypomethylated mESC show aberrant H3K27me3 distribution

**Fig. 1:**
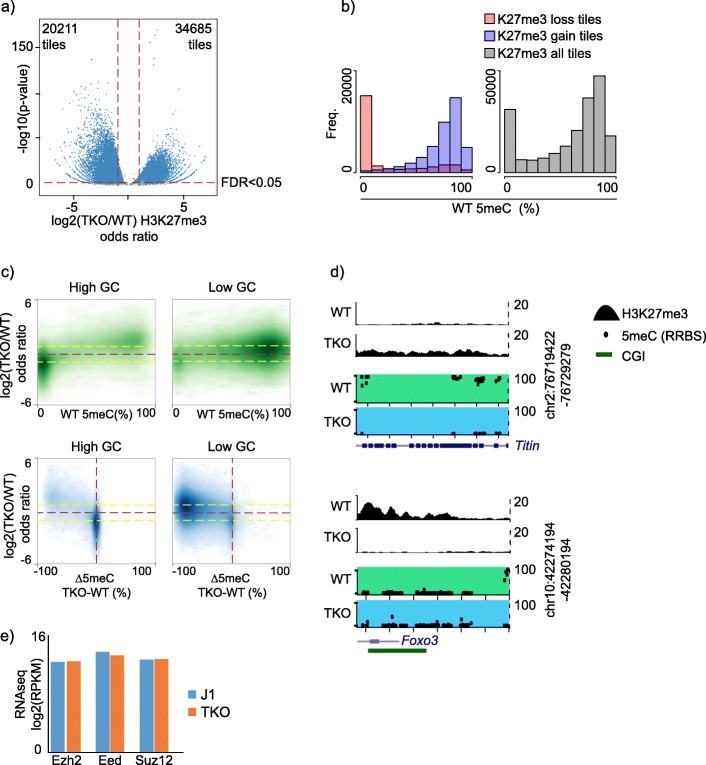
Hypomethylated mESC show aberrant H3K27me3 distribution. **a** Genome-wide volcano plot over 1-kb tiles showing redistribution of H3K27me3 in TKO mESC (*n* = 1). **b** Histograms of RRBS DNA methylation in parental wild-type mESC (*n* = 1) for the indicated H3K27me3 1-kb tiles. **c** Upper: scatter plots of parental wild-type mESC DNA methylation versus log2 fold changes in H3K27me3 for high and low CpG density 1-kb tiles. Lower: scatter plots of differential DNA methylation versus log2 fold changes in H3K27me3 for high and low CpG density 1-kb tiles. **d** Browser images of representative H3K27me3 gain (upper) and H3K27me3 (loss) regions, *Titin* and *Foxo3* genes respectively. **e** RNAseq data in wild-type and TKO ES cells (*n* = 3) showing no major changes in core Polycomb component transcripts Ezh2, Eed and Suz12. See Additional file Table S3 for replicate metrics

To examine the impact of major depletion of 5meC on PRC targeting, we re-analysed publicly available datasets derived from triple knockout mESC lacking three DNA methyltransferases—*Dnmt1, Dnmt3a* and *Dnmt3b* (TKO) [40]. Following multi-omics re-analysis of H3K27me3 ChIPseq and DNA methylation sequencing data (whole-genome bisulfite sequencing (WGBS) and reduced representation bisulfite sequencing (RRBS)), we partitioned the mouse genome into non-overlapping 1 kb tiles [20, 41–43]. We observed that H3K27me3 was severely disrupted in TKO cells: H3K27me3 is lost in 20,211 tiles (overlapping 2943 bivalent promoters) and gained in 34,685 tiles (overlapping 48 bivalent promoters) (Fig. 1a). We related these alterations to parental wild-type mESC DNA methylation and, consistent with other reports, we found that 5meC levels partitioned into two states: lowly (< 10%) or highly methylated (> 80%) (Fig. 1b, right) [20, 44]. Using this approach, we found that TKO-specific H3K27me3 loss regions were poorly methylated in the parental wild-type mES genome (Fig. 1b). In contrast, TKO-specific H3K27me3 gain regions were highly methylated in parental DNA—these reciprocal associations demonstrate, similar to somatic cells lacking 5meC, that H3K27me3 and 5meC co-localisation was relatively rare and loss of 5meC was closely coupled to H3K27me3 redistribution in mESC (Fig. 1b).

H3K27me3 deposition has a preference for GC-rich unmethylated DNA, thought to be mediated in part by the GC-binding CXXC domain of KDM2B which recruits PRC2 through PRC1 [5, 15]. In addition, 5meC is more abundant at lower GC content loci [29]; therefore, we partitioned genomic 1 kb tiles into high and low GC content. In addition to being poorly methylated, tiles where TKO specifically loses H3K27me3 were more GC rich (Fig. 1c). In contrast, besides association with high 5meC levels, TKO-specific H3K27me3 ‘gain’ tiles were of lower GC content (Fig. 1c) [35]. For example, we show a genome browser depiction of mouse *Titin*, which had high wild-type 5meC and lacked H3K27me3 in wild-type mESC. In TKO cells, *Titin* acquired de novo H3K27me3 and was DNA hypomethylated (Fig. 1d). In contrast, the Polycomb target gene *Foxo3*, lacked 5meC and lost most detectable H3K27me3 in TKO cells (Fig. 1d). Importantly, RNAseq data indicated that Polycomb core component expression (Ezh2, Eed and Suz12) was unchanged in TKO cells (Fig. 1e) [32]. Taken together, these experiments showed that hypomethylated mESC have a severely disrupted PRC distribution, which is likely determined by the GC content and DNA methylation status of the parental wild-type genome.

### Identification of two distinct classes of bivalent gene promoters

**Fig. 2:**
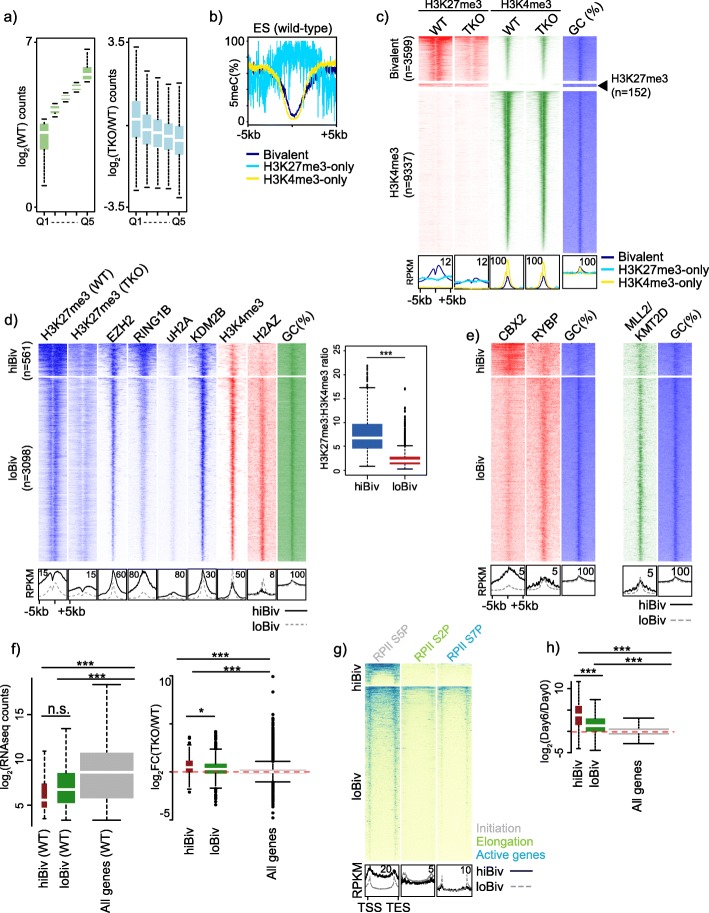
Identification of two classes of bivalent promoters. **a** Boxplots showing H3K27me3 levels in parental wild-type ES quintiles of 1-kb tiled regions (left) and log2-fold changes in H3K27me3 in the indicated quintiles (right). **b** Average plots of DNA methylation (WGBS) in wild-type mESC (*n* = 1) across gene promoters defined as bivalent, HK27me3-only or H3K4me3-only. **c** Heatmap of H3K27me3 and H3K4me3 ChIPseq (*n* = 1) in bivalent, H3K27me3 only and H3K4me3 only regions defined in wild-type mESC. Bottom: average profiles of heatmap data. **d** *k*-means (*k* = 2) clustering of histone modifications (*n* = 1) over bivalent regions in shown in 2c. Bottom: average profiles of heatmap data. Boxplot of mean ChIPseq signal ratios for H3K27me3 and H3K4me3 over hiBiv and loBiv regions. Student’s *t* test used to compare two groups of ratios. **e** ChIPseq heatmap of indicated chromatin-associated factors over hiBiv and loBiv (*n* = 1). Bottom: average profiles of heatmap data. **f** Left: wild-type mESC expression (RNAseq, *n* = 3) of hiBiv and loBiv associated genes compared to all genes; right: log2-fold expression changes between TKO and wild-type mESC (RNAseq, *n* = 3) of hiBiv, loBiv associated genes compared to all genes. **g** ChIPseq heatmap of indicated RPII forms (*n* = 1) over hiBiv and loBiv. Bottom: average profiles of heatmap data. **h** Log2-fold expression changes between activin-induced differentiation and wild-type mESC (RNAseq, *n* = 2) of hiBiv, loBiv associated genes compared to all genes. See Additional file Table S3 for replicate metrics

Given the redistribution of H3K27me3 in hypomethylated mESC, we next asked if pre-existing wild-type H3K27me3 levels had any bearing on its loss in TKO cells. We partitioned wild-type H3K27me3 ChIPseq data into quintiles and then assessed TKO H3K27me3 alterations, demonstrating that the highest wild-type quintile exhibited the most pronounced TKO H3K27me3 loss (Fig. 2a). Conversely, lower wild-type H3K27me3 level quintiles showed a moderate skew towards TKO H3K27me3 gain (Fig. 2a). The majority of H3K27me3 enriched promoters are also marked by H3K4me3 in mES [30], so we next focused our attention on promoters classified as bivalent, H3K27me3-only or H3K4me3-only.

First, we examined DNA methylation across bivalent, H3K27me3-only and H3K4me3-only promoters and observed that H3K4me3 presence was negatively associated with DNA methylation at the centre of promoters and positively associated remote from promoter centres (Fig. 2b, Additional file 1: Figure S1). Bivalent marks have been compared between wild-type and TKO cells [36]. In contrast to promoter annotations used previously, we used a robust annotation of promoters based on multiple (*n* = 8) wild-type mESC [45]. We found a distinct loss of H3K27me3 from bivalent promoters in TKO cells (Fig. 2c). Interestingly, H3K27me3-only regions showed little change in TKO (see average profiles); therefore, we focused on bivalent promoters. Notably, H3K4me3 is unchanged in TKO cells (Fig. 2c). From our analysis, ChIPseq signals for H3K27me3 and H3K4me3 over bivalent promoters existed as a ‘signal’ continuum where the breadth of signal can vary from less than 0.5 kb to greater than 10 kb (Fig. 2c). Therefore, we used an unsupervised machine learning *k*-means approach to ask whether promoters classified as bivalent can be categorised further in terms of related chromatin-modifying and chromatin-associated protein partners. After iterative *k*-means clustering, to optimise the number of clusters to implement, we utilised a *k* value of 2 for further analysis (Additional file 2: Figure S2). We found two distinct bivalent promoter classes: hiBiv promoters had a high H3K27me3:H3K4me3 ratio and higher occupancy by PRC1/2 components; loBiv had a low H3K27me3:H3K4me3 ratio and was relatively enriched for activating marks H3K4me3 and H2AZ (Fig. 2d, Additional file 3: Figure S3, Additional file 4: Figure S4, Additional file 5: Table S1). Given the heterogeneity of PRC1 complexes, we analysed canonical- and non-canonical-specific protein partners [46, 47]. We found that hiBiv was enriched for canonical PRC component CBX2, while loBiv is enriched with non-canonical PRC1.1 RYBP, emphasising the distinct chromatin configurations between these bivalent promoter clusters (Fig. 2e). Recently, it has been shown that *Mll2* knockout mESC lose H3K4me3 and gain H3K27me3 at a subset of bivalent promoters [33]. Mining this dataset showed low levels of MLL2 occupancy in hiBiv and high MLL2 in loBiv, which was consistent with the differential levels of H3K4me3 (Fig. 2e). Finally, Fursova et al. reported a discrete set of mESC loci which retain RING1B in the absence of PCGF1/3/5/6 [48]. We classified these loci as either hiBiv or loBiv and found an enrichment for hiBiv regions which is consistent with the finding that these retained loci are embedded in broad Polycomb chromatin domains (Additional file 6: Figure S5) [48].

One prediction from the differing cluster chromatin compositions is that loBiv genes may be more compatible with transcription. Indeed, analysis of wild-type and TKO RNAseq data showed that loBiv bivalent genes were more highly expressed (albeit at relatively low levels) (Fig. 2f). Interestingly, examination of expression changes in TKO mESC showed that both bivalent clusters are upregulated (median fold change), with hiBiv showing greater upregulation (Fig. 2f). We therefore asked whether this was reflected in differences in RPII engagement and CTD phosphorylation state. hiBiv was relatively enriched for the paused form RPII-S5P, while loBiv is relatively enriched for the elongating form RPII-S2p and the active form RPII-S7P, which is consistent with the transcription associated with these genes (Fig. 2g). Given that hiBiv associated transcription is lower than loBiv in mESC, we asked whether these genes are permissive to transcription during directed differentiation. Using RNAseq data from undifferentiated and activin-induced mESC, we found that both clusters were upregulated in differentiated cells, with hiBiv showing greater transcriptional output (Fig. 2h). Finally, we tested whether stratification of bivalent genes using this approach yielded functional differences in associated gene functions. We found significantly enriched gene sets involved in RPII transcription (hiBiv) and regulation of multicellular organismal process (loBiv) (Additional file 7: Figure S6). Taken together, we have identified two distinct classes of bivalent promoters based on their chromatin configuration, RPII-CTD modification state and permissiveness to transcriptional activation.

### Loss of long-range interactions from hiBiv genes

**Fig. 3:**
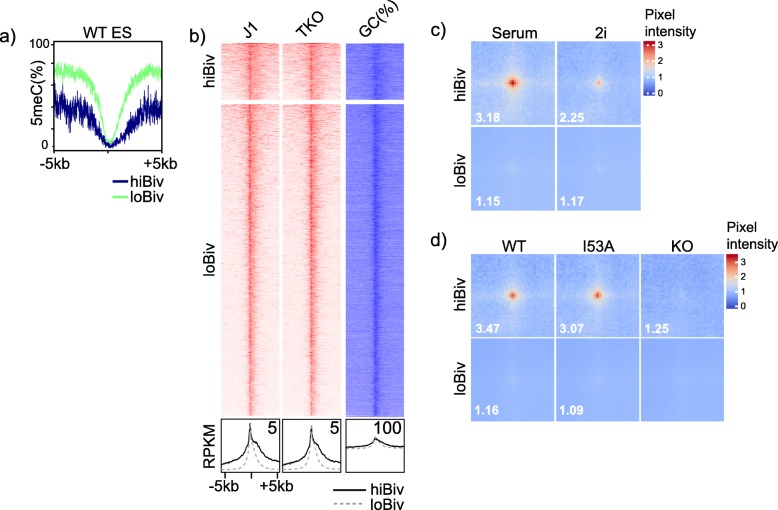
HiBiv chromatin compaction is controlled by DNA methylation and PRC1. **a** Average plots of DNA methylation (WGBS, *n* = 1) in wild-type mESC across gene promoters defined as hiBiv and loBiv. **b** DNaseI-seq (*n* = 2) heatmap over clusters hiBiv and loBiv. Bottom: average profiles of heatmap data. **c** Averaged interaction ‘pileups’ between hiBiv and loBiv in Hi-C data (*n* = 2) from serum and 2i cultured cells. Value of the centre pixel is shown in the bottom left corner of each heatmap. Pixel scale on right. **d** Averaged interaction ‘pileups’ between hiBiv and loBiv in Hi-C data from wild-type (*n* = 4), RING1B I53A (*n* = 2) and RING1B KO (*n* = 2) cells. Value of the centre pixel is shown in the bottom left corner of each heatmap. Pixel scale on right. See Additional file Table S3 for replicate metrics

Redistribution of H3K27me3 and 3D genome reorganisation in mESC cultured in 2i conditions (presence of MAPK and GSK3 inhibitors, PD0325901 and CHIR99021 respectively) can be blocked by constitutive Dnmt expression prior to 2i conversion [49]. Reorganisation of H3K27me3 in TKO mESC may be accompanied by alterations in chromatin accessibility and compaction. Given the differential chromatin configuration of hiBiv and loBiv, it is possible that differences in higher-order chromatin structure exist between the classes. First, analysis of wild-type DNA methylation across hiBiv and loBiv showed low levels of methylation centrally. In contrast, approximately 2 kb from the regions centres we observed higher levels of DNA methylation most noticeably at loBiv (Fig. 3a). Next, we analysed DNaseI-seq (proxy for chromatin accessibility) data from wild-type and TKO mESC. TKO-specific DNaseI hypersensitive sites (DHS) are found at GC-poor regions distal from transcription start sites (TSS) [50]. Consistent with this, we found no clear changes in enzymatic sensitivity between wild-type and TKO mESC over a range of 10 kb in either cluster (Fig. 3b). Of note, hiBiv appeared to be more accessible than loBiv in regions surrounding the peak centre using this assay; however, this was not associated with DNA hypomethylation (comparing J1 to TKO). Indeed, higher accessibility of hiBiv remote from cluster centres is associated with lower levels of DNA methylation.

An alternative index of chromatin structure is biophysical inter-region interactivity which can be measured using chromosome conformation capture (3C) techniques. We analysed Hi-C data (‘all versus all’ interaction contacts) using pileup averages of intra-chromosomal interactions in mESC and found many more contacts in hiBiv than loBiv under serum conditions, which implied lower levels of compaction in loBiv and was consistent with the associated PRC1 components (Figs. 2d and 3c). mESC cultured in 2i are thought to more closely represent cells of the inner cell mass and are globally DNA hypomethylated compared to serum cultured cells [51]. Interestingly, we found reduced frequency of hiBiv long-range contacts in transit between serum and 2i (Fig. 3c). To test the role of PRC in chromatin conformation at different clusters, we compared wild-type, E3 ligase null *Ring1b* and *Ring1b* knockout mESC average pileups. We found that average pileup contacts are dependent on the presence of RING1B protein but not its E3 ligase activity (Fig. 3d). Taken together, these findings suggested a functional difference between hiBiv and loBiv promoters based on higher order chromatin structure.

### Dynamics of hiBiv and loBiv promoters in early embryonic development

**Fig. 4:**
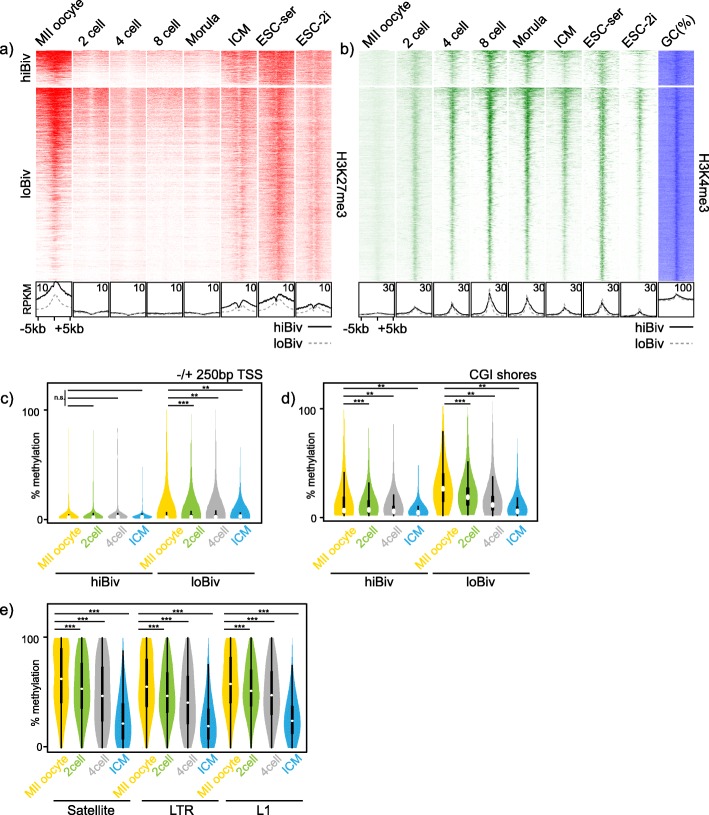
Developmental dynamics of Polycomb and DNA methylation. **a, b** ChIPseq heatmap profiles for H3K27me3 (red) and H3K4me3 (green) in oocyte, early mouse cleavage developmental stages and mESC (serum and 2i conditions). Bottom: average profiles of heatmap data. H3K27me3 replicates: oocyte (*n* = 3), 2 cell (*n* = 3), 4 cell (*n* = 3), 8 cell (*n* = 3), morula (*n* = 3), ICM (*n* = 2), ESC serum [4] and ESC-2i (*n* = 1). H3K4me3 replicates: oocyte (*n* = 4), 2 cell (*n* = 3), 4 cell (*n* = 3), 8 cell (*n* = 3), morula (n = 3), ICM (*n* = 2), ESC serum [2] and ESC-2i (*n* = 1). **c** Violin plots of WGBS DNA methylation levels, adjacent to TSS, in the indicated samples for hiBiv and loBiv. **d** Violin plots of WGBS DNA methylation levels, at CGI shores, in the indicated samples for hiBiv and loBiv. **e** Violin plots of WGBS DNA methylation levels at repetitive sequences in the indicated samples for hiBiv and loBiv. Replicates (**c**–**e**): oocyte (*n* = 6), 2 cell (*n* = 9), 4 cell (*n* = 9) and ICM (*n* = 5). See Additional file Table S3 for replicate metrics

mESC are isolated and derived from the inner cell mass (ICM) of early mouse blastocysts and are thought to reflect the pluripotent state of this developmental stage [52]. Using the clustering sets defined from chromatin marks in mESC (Fig. 2d), we mapped H3K27me3 and H3K4me3 in oocytes and early cleavage stages of the mouse zygote [23]. We found that these chromatin marks were highly dynamic over this narrow window of development (Fig. 4a, b). H3K27me3 was highly abundant in mouse oocytes and this was dramatically reduced post-fertilisation before recovery to higher levels in the blastocyst ICM (Fig. 4a). Similar to the mESC signature, ICM H3K27me3 was lower in loBiv than hiBiv (Fig. 4a). H3K4me3 had a reciprocal pattern to H3K27me3: signal was absent in oocytes followed by establishment at the two-cell stage through to morula stages during which H3K27me3 was very low (Fig. 4a, b). Consistent with the idea that mESC reflect their tissue of origin, the ICM and mESC patterns for both marks were globally highly comparable (Additional file 8: Figure S7). Of interest, was the fact that conversion of mESC from serum to 2i conditions yielded H3K27me3/H3K4me3 profiles more similar to the ICM than mESC cultured in serum (Fig. 4a, b).

To relate the dynamic remodelling of bivalency in early development to DNA methylation, we examined WGBS data in oocytes and early embryonic stages (two-cell, four-cell and ICM). We determined that DNA methylation centred on the TSS of hiBiv regions was very low at the stages analysed, in contrast to loBiv, which was moderately methylated (Fig. 4c). In general, important regulatory sequences remote from TSS, termed CGI shores, had higher levels of methylation which was depleted as embryogenesis proceeded (Fig. 4d) [53]. Importantly, the relationship between promoter class (hiBiv or loBiv) and DNA methylation is consistent between early embryos and ES cells (Figs. 3a and 4a–d). Interestingly, repetitive DNA methylation which was high in female germ cells is reduced in two-cell and four-cell stages followed by a dramatic reduction in the ICM (Fig. 4e). These observations were consistent with the global DNA hypomethylation during early mouse embryogenesis [54]. Therefore, global reduction in DNA methylation between female germ cells and early embryonic cleavages correlated with remodelling of H3K27me3 at promoters. Notably, this relationship was not observed in the ICM as here DNA methylation was relatively low while H3K27me3 recovered (Fig. 4a, e).

### H3K27me3 redistribution is generally associated with DNA hypomethylation and is reversible

**Fig. 5:**
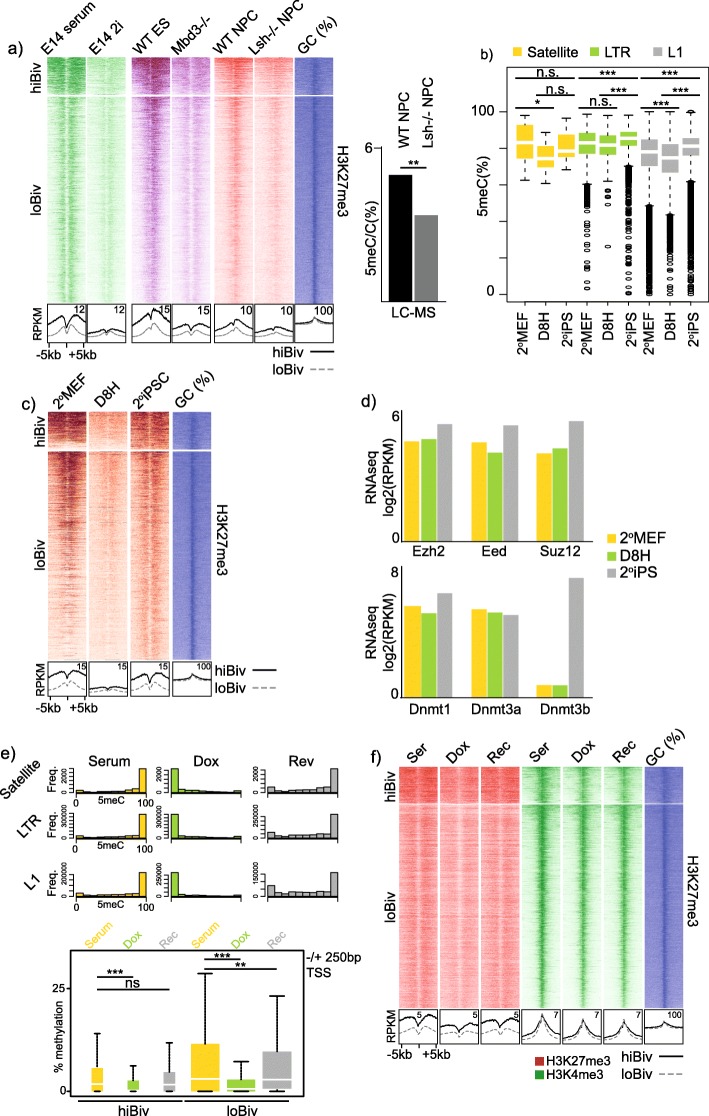
DNA methylation dependent Polycomb targeting is common and reversible. **a** Left: H3K27me3 ChIPseq heatmap profiles for hiBiv and loBiv in the indicated cell types and associated mutants. Bottom: average profiles of heatmap data. Replicates: E14 serum/2i (*n* = 1), ES WT/Mbd3−/− (*n* = 2) and NPC WT/Lsh−/− (*n* = 1). Right: 5meC liquid chromatography mass spectrometry (LC-MS) comparing wild-type and *Lsh−/−* NPC (*n* = 3). **b** Repeat class DNA methylation (WGBS) boxplots during iPSC reprogramming. Replicates: 2^o^MEF (*n* = 8), D8H (*n* = 11), 2^o^IPS (*n* = 8). **c** H3K27me3 ChIPseq (*n* = 1) heatmap profiles over hiBiv and loBiv during iPSC reprogramming. Bottom: average profiles of heatmap data. **d** RNAseq expression barplots for Polycomb components (top) and DNA methylation components (bottom) during iPS cell reprogramming. 2^o^MEF: secondary reprogrammed MEFs; D8H: OSKM transduced MEFs in high doxycycline after 8 days; 2^o^iPSC: secondary reprogrammed induced pluripotent stem cells. **e** Histograms (Satellite, LTR & L1) and boxplots (TSS) of WGBS (*n* = 1) during DNA methylation manipulation by repression and re-activation of Dnmt1 expression in mESC. **f** H3K27me3 ChIPseq (*n* = 1) heatmap profiles over hiBiv and loBiv during DNA methylation manipulation. Bottom: average profiles of heatmap data. See Additional file Table S3 for replicate metrics

While TKO mESC are a widely utilised mouse model of DNA hypomethylation, we next tested other DNA hypomethylation systems based on different mutations and culture conditions for appropriate H3K27me3 localisation. Using hiBiv and loBiv as the bivalent PRC reference framework, we analysed ChIPseq datasets from mESC cultured in 2i, *Mbd3*^*−/−*^ KO cells and *Lsh*^*−/−*^ neural progenitor cells (NPC): ES 2i cells are globally hypomethylated, *Mbd3*^*−/−*^ ES and *Lsh*^*−/−*^ NPC cells are depleted of 5meC at repetitive sequences (Fig. 5a) [51, 55]. In agreement with our findings in TKO cells, all the hypomethylated cell type conditions showed a pronounced H3K27me3 depletion from both clusters, with a greater effect on hiBiv regions (Fig. 5a).

Differentiated cell types can be reprogrammed to induced pluripotent stem cells (iPSC) in the presence of specific transcription factors—for example *OCT4*, *SOX2*, *KLF4* and *C-MYC* (OSKM) [47]. We leveraged publically available datasets for H3K27me3 ChIPseq and WGBS from ‘Project Grandiose’, where mouse embryonic fibroblasts (MEF) were passed through two rounds of iPSC reprogramming [51, 56]. Notably, we found that global methylation was remodelled, prominently at repetitive sequences midway through the secondary reprogramming stage and was re-established in the secondary induced pluripotent stem cell (iPSC) population (Fig. 5b). Therefore, we reasoned that if DNA methylation is dynamic during secondary reprogramming, that H3K27me3 would also show temporal alterations during these cellular transitions. Indeed, ChIPseq from the equivalent reprogramming phases indicated that promoter H3K27me3, particularly hiBiv, showed similar reprogramming dynamics to DNA methylation (Fig. 5b, c). H3K27me3 localisation alterations could not be simply explained by changes in core Polycomb component expression or major changes in Dnmt expression between secondary MEF and midway through reprogramming (D8H, day 8 iPSC in high doxycycline) (Fig. 5d).

We next decided to test whether the reversible nature of H3K27me3/5meC can be recapitulated without germline transmission and viral OSKM integration. In contrast to previous Dnmt complementation experiments in DNA methylation mutant mESC [36], we utilised a system where the maintenance methyltransferase Dnmt1 is under the control of a TET-off regulator—presence of doxycycline (Dox) leads to highly tuneable acute Dnmt1 downregulation followed by DNA hypomethylation without Dnmt1 deletion [57]. We performed WGBS on *Dnmt1*^*tet/tet*^ mESC under the following conditions: serum, serum plus Dox (7 days) and serum upon Dox withdrawal (7 days). We examined 5meC at repetitive sequences and found robust reduction of 5meC in the presence of Dox, which largely recovered after Dox withdrawal (Fig. 5e). In addition, although hiBiv and loBiv TSS had low 5meC levels in serum, the dynamics of repetitive DNA 5meC changes were similar at TSS—thus, in this system, DNA methylation was largely reversible independent of germline transmission and OSKM integration (Fig. 5e). Given our findings in MEF to iPSC reprogramming, we performed H3K27me3 ChIPseq at the equivalent time points and found that DNA hypomethylation was coincident with prominent H3K27me3 loss from hiBiv (Fig. 5f). Importantly, we found that H3K27me3 deposition was reversible in this system as its levels recovered to untreated serum levels subsequent to Dox withdrawal. Notably, H3K27me3 loss from bivalent promoters had no major impact on H3K4me3 (Fig. 5f).

### hiBiv are targets for DNA hypermethylation in cancer

**Fig. 6:**
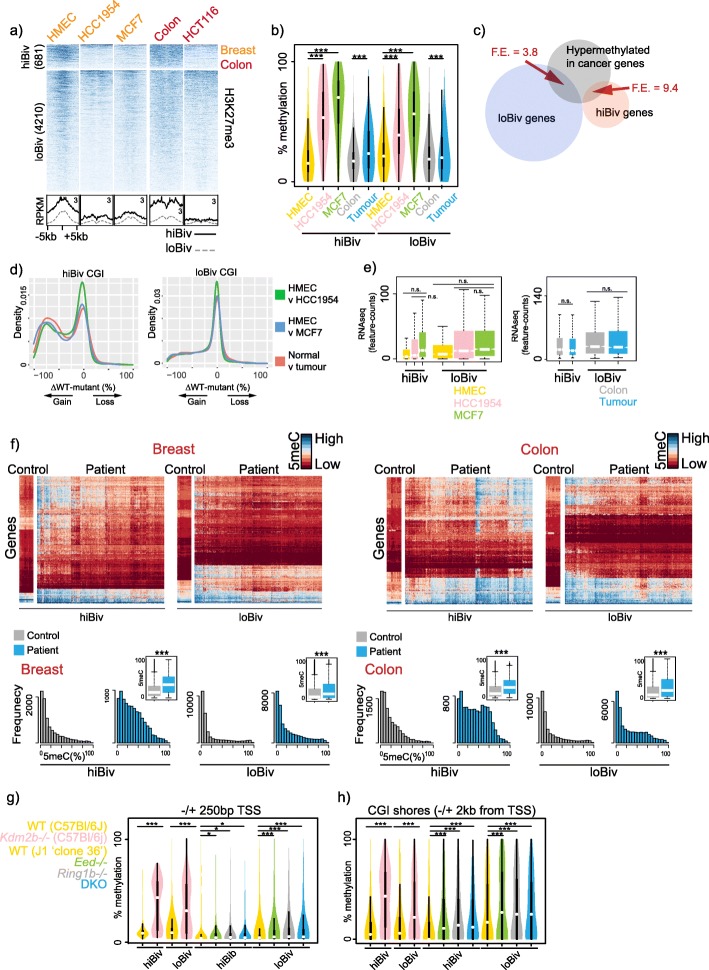
DNA Hypermethylation of hiBiv in cancer. **a** Normal and cancer H3K27me3 ChIPseq (*n* = 1) heatmap profiles. Bottom: average profiles of heatmap data. **b** Violin plots of absolute DNA methylation (WGBS) levels genome-wide in the indicated samples for hiBiv and loBiv. Replicates: HMEC (*n* = 10), HCC1954 (*n* = 15), MCF7 (*n* = 1), colon (*n* = 3), tumour (*n* = 3). **c** Venn diagram of genes frequently methylated in cancer and genes associated with human hiBiv and loBiv. F. E = fold-enrichment over random gene selection. **d** Kernel density plots of differential methylation at human hiBiv and loBiv. **e** RNAseq boxplots for normal and cancer samples in the indicated clusters. Replicates: HMEC (*n* = 3), HCC1954 (*n* = 4), MCF7 (*n* = 3), colon (*n* = 18), tumour (*n* = 18). **f** Pan-Cancer Atlas Infinium 450K DNA methylation data derived from breast and gastrointestinal tissues. Upper: mean gene promoter unclustered methylation heatmaps; lower: histogram distribution of promoter associated individual CpG methylation and inset boxplots with Fisher’s exact test. Breast dataset: controls (*n* = 86), patients (*n* = 780); colon dataset: controls (*n* = 33), patients (*n* = 291). **g** Violin plots of absolute DNA methylation (WGBS) levels, adjacent to TSS, in the indicated samples for hiBiv and loBiv. **h** Violin plots of WGBS absolute DNA methylation levels, at CGI shores, in the indicated samples for hiBiv and loBiv. Replicates (**g**, **h**): WT-C57Bl/6 J (*n* = 1), Kdm2b−/− (*n* = 1), WT-J1 (*n* = 1), Eed−/− (*n* = 1), Ring1b (*n* = 1) and DKO (*n* = 1). See Additional file Table S3 for replicate metrics

Employing a similar approach used to predict murine bivalent promoters, we generated a robust set of bivalent promoters in hESC. Using H3K4me3 and H3K27me3 ChIPseq from hESC, we grouped bivalent promoters into two *k-*means clusters and, similar to mouse, hiBiv had a high H3K27me3:H3K4me3 ratio while loBiv had a low ratio (Additional file 9: Figure S8; Additional file 10: Table S2). As for mouse, stratification of human bivalent promoters in this manner yielded differential enrichment of gene ontologies (Additional file 11: Figure S9). Given our finding that DNA hypomethylation is associated with H3K27me3 redistribution in a variety of mouse systems, we tested whether this finding is conserved in cancer cell line models and solid tumours. Strikingly, we observed a pronounced loss of H3K27me3 from hiBiv when comparing human mammary epithelial cell (HMEC) to breast cancer cell lines or normal colon to HCT116 colorectal cancer cells (Fig. 6a, Additional file 12: Figure S10). H3K27me3 depletion from loBiv was not as pronounced, consistent with mouse data where we demonstrated that bivalent promoters with the highest wild-type H3K27me3 levels were most depleted of the mark under DNA hypomethylation conditions (Fig. 2a, Additional file 12: Figure S10). A hallmark of cancer is frequent hypermethylation of CGI promoters of cell cycle regulation genes and it has been noted that these targets are often bivalent in pluripotent mESC [37, 38]. Therefore, we analysed WGBS from the equivalent cell lines and tissues and determined that hiBiv regions were more highly methylated in breast cancer cell lines than HMEC and to a lesser degree in colonic tumour compared to normal tissue (Fig. 6b). LoBiv promoters were less hypermethylated in cancer cell lines relative to HMEC and in colon tumour compared to normal colonic tissue (Fig. 6b). Next, we compared hiBiv and loBiv associated genes with frequently hypermethylated cancer gene promoters and found ~ 2.5 fold greater enrichment of hiBiv promoters within this set compared to loBiv (Fig. 6c). Additionally, differential methylation analysis also showed greater hypermethylation in hiBiv relative to loBiv (Fig. 6d).

Previously, it was shown that frequently hypermethylated genes are already repressed in originating pre-cancerous tissues [58]; therefore, we analysed RNAseq data from breast and colon samples. We found that genes associated with hiBiv and loBiv promoters, which have depleted H3K27me3 and higher 5meC, were not significantly altered in expression (Fig. 6e). This implies that one transcriptional repression mechanism (DNA methylation) may be compensating for the loss of a distinct repressive mechanism (Polycomb). To expand on these findings, we mined Pan-Cancer Atlas Infinium 450K DNA methylation data parsed for breast tissues and gastrointestinal tract. Mean CpG promoter methylation was computed for controls and patients and hierarchical clustering heatmaps indicate qualitative differences in hypermethylation between hiBiv and loBiv patients (Fig. 6f; Additional file 13: Figure S11; Additional file 14: Figure S12). Indeed, analysis of unique individual CpG methylation showed a greater skew towards hypermethylation in hiBiv compared to loBiv (Fig. 6f). We also report similar trends in gastrointestinal tumours (Fig. 6f). Importantly, we found little evidence of DNA hypermethylation at promoters that are characterised as H3Kme3-only or H3K27me3-only, supporting the idea that bivalent genes are more prone to DNA hypermethylation and in particular hiBiv promoters (Additional file 15: Figure S13; Additional file 16: Figure S14).

**Fig. 7:**
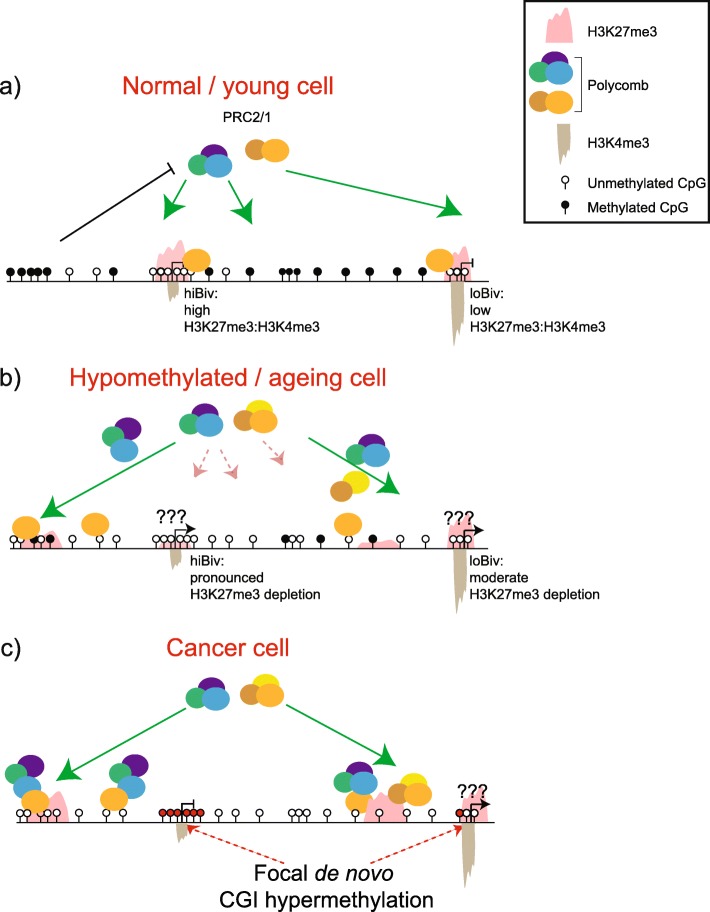
Model for cancer DNA hypermethylation. **a** Polycomb complexes target H3K27me3 to unmethylated CGI (green arrows) enriched for variable levels of H3K4me3. H3K27me3 is occluded from methylated interspersed CpG (black bar), e.g. repetitive elements/satellite DNA. Such normal Polycomb targets are transcriptionally silenced. **b** Ageing cells and cells undergoing the early events of transformation become globally hypomethylated at repetitive sequences. Polycomb H3K27me3 is redirected (most pronounced from hiBiv loci) to the high burden of unmethylated interspersed CpG. It is unclear during this phase if hiBiv promoters are expressed. **c** At later stages of cellular transformation and tumours, unprotected CGI are targets for retained de novo DNA methyltransferase activities—which is more prominent at hiBiv loci

Global hypomethylation is a common feature of cancers and may explain, in part, depletion of H3K27me3 from cognate target sites [21, 33]. In addition to DNA hypomethylation, abnormal Polycomb expression and/or function has been described in many malignancies. Moreover, investigation of the impact of Polycomb loss on mESC DNA methylation has been limited to a reduced genomic fraction (4 kb over mouse TSS) [32]. Thus, we focused on cultured mESC lacking key factors involved in the maintenance of PRC-mediated repression (KDM2B, EED or RING1B). Consistent with previous findings in *Kdm2b*^*−/−*^ mESC [15], where KDM2B is enriched at the majority of wild-type CGIs, we detected hypermethylation at both hiBiv and loBiv in 500-bp windows across TSS of the associated genes (Fig. 6g). Surprisingly, although KDM2B is involved in non-canonical PRC1 repression and targeting to CGIs, we found little evidence of hypermethylation in mESC lacking the PRC1 core factor RING1B (Fig. 6g). Furthermore, we observed little hypermethylation in mESC lacking the PRC2 components EED and RING1B (or in a *Ring1b*^*−/−*^/*Eed*^*−/−*^ double knockout) at either cluster. CGI shores and their methylation states can contribute to tissue-specific expression patterns, are highly conserved in mouse and are frequently hypermethylated in cancer in contrast to core CGI sequences [53]. Thus, we extended our mESC PRC knockout analysis to CGI shores 2 kb from TSS. Surprisingly, unlike core TSS sequences, we detected hypermethylation of CGI shores in PRC mutant mESC specific to hiBiv (Fig. 6h). In contrast, wild-type loBiv CGI shores were already methylated and thus methylation increases in PRC mouse knockouts were minimal. In summary, we found that hiBiv-associated promoters are more susceptible to hypermethylation in cancer (Fig. 7), and that unlike deletion of non-canonical Kdm2b in mESC, deletion of canonical PRC components was associated with CGI shore hypermethylation.

## Discussion

Here, combining systematic and comprehensive mapping of H3K27me3 and DNA methylation with chromatin conformation and transcription, we report on Polycomb reorganisation in states of DNA hypomethylation. We determined that H3K27me3 localisation is widely dependent on DNA methylation and H3K4me3 levels. Accordingly, Polycomb occupancy and DNA methylation interplay is determined by specific chromatin configurations and these relationships are pervasive in stem cells, mouse development and cancer.

hiBiv promoters (enriched in Polycomb components with high degree of chromatin contacts) exhibit reduced contacts in DNA hypomethylated states and Polycomb mutants. Many of these are Homeobox genes that exhibit reduced chromatin compaction by FISH under the same conditions [49]. Thus, the developmental occurrence of bivalency and its participation in the formation of higher-order chromatin structures maybe linked with de novo methylation in early development [23, 59, 60]. Relevant to this is our finding that H3K4me3 is prominent during DNA demethylation in the early mouse embryo. Such a scenario can be envisaged as global demethylation triggering H3K27me3 redistribution, which renders gene promoters permissive to H3K4me3 accumulation and subsequent initiation of zygotic gene activation (ZGA). Notably, during ZGA, H3K27me3 is depleted from hiBiv and loBiv equally, while H3K4me3 accumulates to higher levels at loBiv regions, in agreement with our observation that loBiv regions have a higher potential for a positive transcriptional output.

A previous study demonstrated that complementation of de novo methyltransferase activities in DKO (*Dnmt3a*^*−/−*^*, Dnmt3b*^*−/−*^) hypomethylated mESC partially restores a wild-type chromatin state in terms of H3K27me3 localisation [36]. Global loss of DNA methylation in our models (mESC models: 2i and *Mbd3−/−*; OSKM reprogramming intermediates and *Lsh−/−*NPCs) is accompanied by striking H3K27me3 depletion from target sites at highly H3K27me3 enriched hiBiv regions. Unlike the DKO complementation rescue experiments, this state is fully reversible with re-expression of DNMT1 or reprogramming of differentiated cells to iPS cells, which is likely to be a critical mechanism during phases of DNA demethylation and remethylation in early zygote and germ cells [61, 62]. A molecular explanation for the difference between DKO ESC and models explored here is unclear. DKO cells are null for Dnmt3a/Dnmt3b while our reversion experiments retain all three DNA methyltransferases and the fibroblast reprograming is transmitted through the germline—thus providing a potential explanation but further work may be informative.

Despite many advances, it is still unclear how de novo DNA methylation at bivalent CGIs is targeted in cancer [63]. The number of hESC bivalent chromatin CGIs do not fully account for the variability of CGI hypermethylation observed in breast and colon cancers. ESC hiBiv CGIs, enriched in canonical PRC1 components and much reduced H3K4me3, that are preferentially hypermethylated in cancer add clarity to this process. De novo DNA methylation is mediated by the histone modification state of its target sequences and is inhibited by the presence of H3K4me3 [64, 65]. Global DNA hypomethylation in cancer and subsequent H3K27me3 hiBiv depletion may be sufficient for aberrant DNMT3B activity to promote hypermethylation at these regions. This is likely not the case for loBiv bivalent CGI as higher levels of H3K4me3 will inhibit potential de novo methyltransferase activity [66]. In a DNMT3B over-expression model, low expression H3K27me3 enriched CGIs were the prime target for hypermethylation while regions enriched for H3K4me3 were protected from DNA methylation gains [66]. A missense mutation (Dnmt3a^D329A^) in the PWWP targeting domain of DNMT3A can result in hypermethylation of CGI in mice particularly at CGI promoters and gene bodies marked by H3K27me3, representing an alternative hypermethylation pathway which may be utilised in specific cancer contexts such as acute myeloid leukaemia [67]. Interestingly a similar mutation identified in a patient with microcephalic dwarfism (Dnmt3a^W326R^) results in CGI methylation in the continued presence of H3K27me3 [39, 67].

We propose that in normal cells spurious de novo DNA methylation at CGI is prevented by opposing DNA demethylation pathways (TET enzymes), PRC1/2 complexes and H3K4me3. Indeed, there is a relative enrichment of 5hmC bivalent promoters in normal cells that are prone to de novo methylation in a mouse model of liver cancer [68]. Interestingly, loss of 5hmC is an early event in this model and precedes the appearance of hypermethylated regions in the end-point cancers. Thus, we can speculate that similar to early mouse development, the order of events is loss of 5hmC protection at hiBiv CGI followed by global hypomethylation redirecting H3K27me3 away from these regions enabling de novo methylation at regions with low levels of H3K4me3. Additionally, our findings potentiate a role for a lack of H3K4me3 in enabling de novo methylation at potentially vulnerable hiBiv CGI. In the future, it may be useful to investigate whether there is also a link between low-level H3K4me3 and de novo DNA methylation at other discrete sets of CGI in cancer.

An alternative model is direct interference with Polycomb function, which also occurs in many cancers and may lead to hypermethylation [69–71]. Our analysis of mESC Polycomb mutants supports a causal association: inactivation of the PRC1.1 variant component Kdm2b leads to significant hypermethylation of hiBiv CGI and shores whereas inactivation of core PRC1/PRC2 components, RING1B and EED respectively, leads to methylation of CGI shores at hiBiv regions. The continued presence of KDM2B in these mutants may protect core hiBiv CGI, and to a lesser degree loBiv CGI, from de novo methylation.

Much interest has centred on hypermethylation of genes in cancer, where such events may act as drivers of tumourigenesis—assuming susceptible genes are silenced and are involved in key cellular processes including proliferation, apoptosis, senescence, angiogenesis, adhesion and many others [63]. We find little evidence of gene expression changes in hypermethylated hiBiv/loBiv in transformed cell/tissue types. Therefore, H3K27me3 depletion triggered by global hypomethylation in cancer cells may be compensated by focal de novo hypermethylation at H3K27me3-vacated loci—a scenario in which presumably one repressive mechanism replaces another to maintain silencing of these tightly regulated lowly expressed genes whose expression may interfere with cancer phenotypes (Fig. 7) [63].

## Conclusion

A key conclusion from our findings is that classification of bivalent gene promoters requires diligent sub-classification using machine learning approaches based on epigenetic marks. We propose that bivalent promoters should not be considered a ‘blanket’ term but rather that subclasses of bivalency exist in the context of an occupancy continuum. Our novel distinction reported here provides the framework to identify fundamental mechanisms underlying defects in DNA methylation and chromatin state. We and others have previously shown that aberrant 5meC can dramatically impact H3K27me3 distribution in somatic and pluripotent mouse cells [21, 36]. Now, we report consistency in this finding in other DNA hypomethylation systems in higher eukaryotes. We foresee that the application of bivalent gene sub-classification to disease metadata will reveal common themes (analogous to our findings) and disease-specific events perhaps relating to disease pathology (i.e. tumour stage), tissue of origin and degree of DNA hypomethylation or Polycomb mutation load. In summary, this study refines our knowledge of the antagonistic interplay between DNA methylation and Polycomb and the complexity underlying bivalency. Understanding these principles in disease contexts is a priority.

## Materials and methods

### Cell culture

All cell lines were cultured using standard procedures. Wild-type mESC (J1) were adapted to feeder-free conditions and grown on gelatin-coated culture plates in media composed of 15% FCS, GMEM (Gibco), 1% pen-strep, 1% non-essential amino acids, 1% sodium pyruvate, 1% glutamine, 0.1 mM beta-mercaptoethanol and 500 U/mL ESGRO LIF. NPCs were derived from ESCs and cultured as previously described [72]. mESC were split at high density 24 h prior to differentiation, followed by dissociation and PBS washes to remove any traces of differentiation-inhibitory serum. Cells were then cultured in neurodifferentiation media (1:1 mix of Neurobasal (Gibco) and DMEM/F-12 media (Gibco) with 0.5% Neuro-2 Supplement (Millipore), 0.5% B27 Supplement (Gibco), 0.2 mM l-glutamine and 0.1 mM β-mercaptoethanol) and plated onto 0.1% gelatin-coated flasks at a density of 3 × 10^5^ cells per 75-cm^2^ flask area. Media was changed on day 2 of differentiation and then daily thereafter. Neural progenitor cells (10 days) cultures were enriched using two passes of Magnetic Activated Cell Sorting (MACS) with the anti-PSA-NCAM antibody Microbead kit (Miltenyi Biotec; #130-097-859; lot: 5161208294; RRID: AB_2752096). Fluorescence-activated cell sorting (FACS) using anti-PSA-NCAM antibody was employed to assess NPC purity post MACS. For *Dnmt1*^*tet/tet*^ mESC experiments (clone termed ‘Ch9’), cells were cultured as previously described except ESGRO LIF (Millipore) was used at 500 U/mL. For Dnmt1 depletion, doxycycline was added to media daily for 7 days at a concentration of 2μg/mL. For recovery, doxycycline was withdrawn for 7 days. *Dnmt1*^*tet/tet*^ mESC cells were a gift from Richard Chaillet [57].

### ChIPseq

Native ChIPseq was performed as previously described [21]. In brief, soluble chromatin was isolated from nuclei using NBR buffer (85 mM NaCl, 5.5% sucrose, 10 mM Tris, 3 mM MgCl_2_, 1.5 mM CaCl_2_,0.2 mM phenylmethylsulfonyl fluoride, 1 mM dithiothreitol) and micrococcal nuclease (Worthington, Lakewood, NJ, USA) overnight at 4 °C. Clarified soluble chromatin was isolated by centrifugation. Immunoprecipitation was carried out with antibodies specific to H3K4me3 (#07473, Millipore; lot: 2019729; RRID: AB_1977252) and H3K27me3 (#07449, Millipore; lot: DAM1662421; RRID:AB_310624) followed by antibody-optimised washes. Samples from native ChIP were purified using affinity columns (Qiagen, USA), and ChIP was validated using positive and negative control region primer sets. ChIP DNA was treated with RNaseA (Roche Diagnostics, West Sussex, UK) at 37 °C for 1.5 h and with Proteinase K addition at 55 °C for 1 h. ChIP DNA were purified using the QIAquick PCR purification kit (Qiagen). Library DNA was assessed using a Bioanalyzer and quantitated using a Qubit instrument, and sent to Edinburgh Genomics (UK) for Illumina library preparation and sequencing. Crosslinked ChIP was performed for Kdm2b and uH2A (antibodies were a gift from Robert Klose, Oxford) as previously described [10], and barcoded libraries were prepared using NEBNext® Ultra™ DNA Library Prep Kit for Illumina coupled with NEBNext® Multiplex Oligos for Illumina (E7335/E7500, NEB, USA).

### RNAseq

Directional RNA Library Prep Kit for Illumina (E7420, NEB, USA) in conjunction with NEBNext® Multiplex Oligos for Illumina (E7335/E7500, NEB, USA). Libraries were assessed for amplification, size range and quantity using the Bioanalyzer and Qubit. Adaptor barcoded libraries were sequenced using v4 chemistry paired-end strand-specific 50 bp on an Illumina HiSeq 2500 instrument (Illumina, USA) by Edinburgh Genomics (University of Edinburgh) or using a NextSeq 500 instrument by the WTCRF (Edinburgh University).

### Bisulfite sequencing and liquid chromatography mass spectrometry (LC-MS)

Genomic DNA was isolated from cell lines using lysis buffer SETN (0.2% SDS, 5 mM EDTA pH 8.0, 100 mM Tris-HCl pH 8.0 and 200 mM NaCl) and purified by proteinase K, RNase A/T1 treatment followed by phenol:chloroform extraction into TE buffer. Aliquots of genomic DNA were assessed for quality, and whole-genome bisulfite sequencing libraries were prepared and sequenced using a NextSeq 500 instrument by the WTCRF (Edinburgh University).

For LC-MS, 1 μg of DNA was hydrolysed to single nucleotides with 10 units DNA degradase enzyme (Zymo Research) and 2.5 μl DNA degradase buffer (Zymo Research) in a 25 μl reaction. Samples were mixed with 100 μl mass spectrometry-grade methanol and 60 μl mass spectrometry-grade acetonitrile (Sigma), centrifuged at 10,000 rpm for 5 min and the upper 30 μl of supernatant was transferred to mass spectrometry glass vials. Standards for unmodified cytosine, 5mC and 5hmC were ran alongside the samples for comparison. Liquid chromatography samples were separated on a SeQuant ZIC-pHILIC column using a Thermo UltiMate 3000 BioRS with a flow rate of 0.3 ml/min and a gradient of 90% to 5% acetonitrile in 10 min. Eluting peptides were analysed on a Thermo Q Exactive in negative mode, scanning from 300 to 350 m/z at resolution 70 k. AGC target was set to 3 × 106 and maximum ion time 500 ms. Data was analysed using the Xcaliber programme to quantify the area under the peaks. Levels of 5mC were taken as a percentage of total cytosines (i.e. unmodified, methylated and hydroxymethylated).

### Datasets

Next-generation sequencing datasets were obtained from multiple public sources: NCBI (http://www.ncbi.nlm.nih.gov/sra & http://www.ncbi.nlm.nih.gov/geo), EBI (http://www.ebi.ac.uk/ena) and DDBJ (http://trace.ddbj.nig.ac.jp/index_e.html). See Additional file 17 Table S3.

### ChIPseq analysis and data display

ChIPseq data was downloaded from publically available sources and produced in-house. In most cases, NGS data was downloaded in the form of compressed fastq files. Next, library quality was assessed using FastQC tools by sampling 1 million reads and viewing base statistics, per base sequence quality, sequence duplication levels and adaptor contamination. In addition, Picard tools was used with Java in Unix to assess library complexity. In appropriate cases, reads were either 5′ or 3′ trimmed as required to remove adaptors, barcodes, read-through adaptors, read-through barcodes and low sequence quality ends (TrimGalore tool from FastQC suite. Extra settings were: -q 30 –illumina/nextera --stringency 6). Fastq raw data was mapped to the mouse genome (mm10 build) or the human genome (hg38 build) using bowtie v2.2.6: bowtie2 -p 4 -N 1 -t -L 28 --phred33-quals. All mapped bam files were indexed for rapid access using SAMtools bam indexing. For heatmap production, deepTools was implemented. The first step involves calculating genome coverage in mapped bowtie bam output files: bamCoverage –b mapped.bam –bai indexed.bai –normalizeUsingRPKM -p 4 -f 300 --ignoreDuplicates --minMappingQuality 30 –o out.bigwig. Next, a matrix of genome coverage is generated using the coverage file sets of genomic coordinate regions of interest in bed file format using computeMatrix: computeMatrix reference-point --referencePoint center -R regions.of.interest.bed -S out.bigwig(s) --outFileName matrix.file --outFileNameData averages --outFileSortedRegions regions.bed --beforeRegionStartLength 10,000 --afterRegionStartLength 10,000 --binSize 100 --sortRegions no –missingDataAsZero. The final step in heatmap production utilises plotHeatmap: plotHeatmap --matrixFile matrix.file --outFileName heatmap.eps --outFileNameData data_underlying_average_profile --outFileSortedRegions regions.bed --outFileNameMatrix data_underlying_matrix --sortRegions descend --sortUsing mean --averageTypeSummaryPlot mean --missingDataColor 1 --colorMap afmhot_r --zMin 0.0 --zMax 50.0 --heatmapHeight 15.0 --heatmapWidth 7.5 --whatToShow “plot, heatmap and colorbar”. Average profile plots were produced directly from the ‘averages’ output from computeMatrix using the R programming environment. The R ‘t.test’ function was used to assess statistical differences between average profiles. Volcano plots were used to illustrate log2 transformed fold change in TKO/J1 cells for H3K27me3 ChIPseq data. Non-overlapping windows of 1 kb were intersected with ChIPseq read coordinates BEDTools. For each window, log2 transformed (TKO/J1) read depth and –log10 transformed (*p* value from Fisher’s exact test) was computed and plotted using the generic ‘plot’ function in R.

### Bisulfite sequencing and data display

Raw bisulfite sequencing fastq files were trimmed for base call quality and adaptors using TrimGalore (v0.4.1). Bismark (v0.16.3) m-bias plots were produced to plots permit removal of non-native end-repair cytosines, followed by mapping using the following settings: bismark --bowtie2 genome-build (mm10/hg38) --multicore 3 -o output --non_bs_mm − 1 trimmed_read_1–2 trimmed_read_2. Methylation was inferred from bismark_methylation_extractor: bismark_methylation_extractor --gzip --report -o output --multicore 3 --genome_folder genome-build (mm10/hg38) --counts --zero_based --cytosine_report --bedGraph --comprehensive bismark_output. Only cytosines in the CpG context at a minimum depth of five reads were considered for downstream analysis. BEDTools (v2.25.0) was used to overlap CpG methylation data with genomic features (i.e. promoters, CGI shores, repetitive elements) as defined by the UCSC table browser annotations and repeatmasker. Boxplots and violin plots were produced using the boxplot function and the vioplot package in R.

### RNAseq analysis and data display

RNA sequencing fastq files were filtered for base call quality and trimming using FastQC (v0.11.4) and TrimGalore (v0.4.1). Paired end mapping was performed using TopHat (v2.1.1): tophat --no-coverage-search --library-type fr-firststrand -G gene_model(mm10/hg38) -p 8 -o output genome_build(mm10/hg38) fastq_1 fastq_2. Read counts over annotated features (i.e. genes) were computed using featureCounts (v1.6.0) as follows: featureCounts --verbose -g gene_name -p -s 2 -a gene_model(mm10/hg38) -o output tophat_output. featureCount data was imported into R and converted to DESeq objects followed by computation of fold changes using a negative binomial test.

### Correlation between WGBS and H3K27me3 occupancy

To produce the data shown in Fig. 1, processed WGBS and ChIPseq data were read into R memory. Genome-wide analysis: for the 1 kb ‘all windows analysis’, all WGBS data were considered and a histogram was produced in R to reflect the bipartite division of methylation presence (histogram peak signals at either very low methylation (left-hand side of plots) or very high methylation (right-hand side of plots)). Next, we considered ChIPseq 1-kb windows genome-wide with odds ratio scores of > 3 or < 0.33, corresponding to threefold H3K27me3 gain windows and threefold H3K27me3 loss windows respectively in TKO cells. These coordinates were intersected with the WGBS data using BEDTools (v2.25.0) and filtered histograms were plotted.

### Definition of bivalent promoters and *k*-means clustering

Mouse bivalent genes (*n* = 3660) were defined as previously reported [45], where multiple H3K27me3 and H3K4me3 ChIPseq datasets derived from multiple mESC lines were integrated. H3K27me3-only and H3K4me3-only genes were defined in a similar fashion. An unsupervised machine learning algorithm, *k*-means clustering, was implemented on multiple chromatin-related ChIPseq datasets in wild-type mESC using a range of integer values for *k*. One approach to determine the number of clusters is the ‘elbow method’. This involves calculating the sum of squared errors (SSE) for each value of *k*, and plotting a line chart of the SSE value for each value of *k*. A value of *k* = 2 was adopted for cluster definition as values of *k* > 2 extended beyond the elbow point, i.e. increasing the value of *k* > 2 has limited impact on the SSE. To define human bivalent genes, we mapped various H3K27me3 and H3K4me3 ChIPseq datasets from multiple wild-type hESC lines. Next, we carried out *k*-means clustering (*k* = 2) for these datasets using genomic coordinates defined for human bivalent promoters previously described [45].

### Heatmap and average profiles

Heatmaps and average profiles were done using a series of tools within the deepTools suite (v3.2.0). In brief, read coverage tracks (bigwig format) were produced from read alignment files (bam format) using bamCoverage (bamCoverage -b in.bam -o out.bigWig -of bigwig -bl blacklisted_genomic_regions --normalizeUsing RPKM –ignoreDuplicates; matrices underlying heatmaps were generated using computeMatrix (computeMatrix reference-point --referencePoint center -p 4 -S in.bigwig(s) -R regions_of_interest.bed -a 3000 -b 3000 --outFileName output --outFileSortedRegions output_regions); finally heatmaps were plotted using plotHeatmap (plotHeatmap -m in.gz -out output.png --yMin 0 --yMax 25 --dpi 400 --xAxisLabel “from_centre” --plotTitle ‘Title’ --plotFileFormat png –samplesLabel “Sample ID list”).

### DNaseI-seq and Hi-C

DNaseI-seq raw sequencing data was mapped in similar fashion to ChIPseq with minor exceptions. DNase-seq reads were trimmed for adaptors and trimmed to 50 base pairs in length and then mapped: bowtie -p 3 -q --sam -v 3 -m 1 --best --strata genome_build(mm10) - > output.sam. Unmapped reads were trimmed to 25 base pairs remapped as before to rescue short fragments. All reads were merged using SAMtools (v1.3) and peaks were called with MACS2 (v2.1.1). Heatmaps were produced as for ChIPseq. Hi-C data was analysed and average pileups were plotted as described previously [73].

### Enrichment of hypermethylated cancer genes

A panel of frequently hypermethylated genes in cancer was publically available [58]. Clusters 1 and 2 associated gene promoters were overlapped with this set and fold-enrichments were computed compared to iterations of randomly chosen clusters of comparable size from all human genes.

### UCSC browser tracks

Browser tracks for ChIPseq or WGBS were generated by uploading bigwig files into the IGV browser (v2.5.2). Regions of interest were entered as coordinates and images were exported. WGBS and ChIPseq tracks were overlaid in figure production.

### Division of quintiles to define association between wild-type H3K27me3 occupancy and H3K27me3 sensitivity to DNA hypomethylation

Approaches to generate windows of ChIPseq enrichment genome-wide have been previously described. In brief, a custom script was produced in R to divide a given genome into 1-kb windows. Next, bowtie outputs were converted to bed files using BEDTools (v2.25.0), noting mapping metrics for downstream normalisation. Bed files and genomic windows were overlapped using BEDTools (v2.25.0) followed by joining wild-type and paired mutant data into a single database. We next used custom scripts to compute (a) log-transformed fold changes, (b) Fisher’s exact test for testing the null of independence of the database contingency table and (c) odds ratio scores in terms of wild-type and mutant ChIPseq windows, accounting for variations in library read depth. The ChIPseq database was split into quintiles based on wild-type ChIPseq signal, and this split database was used to plot log-transformed mutant/wild-type fold changes within the separate quintiles.

### General bioinformatics tools

SRA Toolkit was employed to extract fastq read files from the Sequence Read Archive format files (.sra) in situations where data was unavailable in compressed fastq format. HOMER was utilised for many core bioinformatics operations including (but not limited to) preparing UCSC format bigWig (.bw) files for browser display and genomic coordinate annotation. Notably, we prepared bigWig files using UCSC tools / BEDTools and we utilised R / BEDTools to manually annotate genomic coordinates to validate our HOMER analyses. To avoid hosting compressed data on internet accessible servers we employed the local genome browser IGV and we used IGVTools to create low memory requirement ChIPseq pileup files in bigWig or tdf format. We utilised DAVID as a comparison for enriched gene ontologies output from REViGO.

### Gene ontology analysis

The Gorilla gene ontology (GO) tool was used to calculate GO term enrichment in hiBiv and loBiv in both mouse and human ESC. Gene lists were submitted to the Gorilla GO server against backgrounds of all unique RefSeq genes for mm10 and hg38; settings: mouse, ‘ontology’ = process, *P* value threshold = 10^−4^; human, ‘ontology’ = process, *P* value threshold = 10^−6^. Gorilla outputs were output to REViGO. Next, the REViGO tool was used to reduce closely related GO term redundancy in the Gorilla outputs. REViGO settings: ‘allowed similarity’ = tiny; ‘numbers associated with GO categories are … ’ = *p* values; ‘database’ = whole UniProt; ‘semantic similarity measure’ = SimRel. R was used to plot the REViGO two-dimensional semantic scatterplots.

### Illumina Infinium 450K data analysis

Publically available DNA methylation data and the pertaining manifest was downloaded from https://gdc.cancer.gov/about-data/publications/pancanatlas followed by parsing into normal and tumour in the Unix environment. Next, extracted data for gastrointestinal tract and breast were intersected with human hiBiv and loBiv using BEDTools. Clustered (hierarchical) mean DNA methylation heatmaps were plotted using R (v3.6.0) encompassing 4 kb over TSS. Histograms were prepared from the same regions of the genome on an individual CpG basis. Differences between normal and tumour datasets were statistically assessed using Student’s *t* test.

**Table:** 

Bioinformatics tool URLs	
R: https://www.r-project.org/	
Bismark: http://www.bioinformatics.babraham.ac.uk/projects/bismark/	
Bowtie: http://bowtie-bio.sourceforge.net/index.shtml	
SRA ToolKit: https://github.com/ncbi/sra-tools	
fastQC: http://www.bioinformatics.babraham.ac.uk/projects/fastqc/	
Picard: http://broadinstitute.github.io/picard/	
SAMTools: http://www.htslib.org/	
deepTools: http://deeptools.readthedocs.org/en/latest/	
BEDTools: http://bedtools.readthedocs.org/en/latest/	
MACs tools: https://github.com/taoliu/MACS	
HOMER tools: http://homer.salk.edu/homer/	
Tophat: https://ccb.jhu.edu/software/tophat/manual.shtml	
featureCounts: http://bioinf.wehi.edu.au/featureCounts/	
IGVTools: https://www.broadinstitute.org/igv/igvtools	
IGV browser: https://www.broadinstitute.org/igv/	
UCSC browser: http://genome.ucsc.edu/	
UCSC tools: http://hgdownload.soe.ucsc.edu/admin/exe/linux.x86_64/	
DAVID: https://david.ncifcrf.gov/home.jsp	
REVIGO tool: https://revigo.irb.hr/	

## Supplementary information

Additional file 1: **Figure S1.** Negative correlation between H3K4me3-only enriched regions and DNA methylation in mouse and human ESC. (Left) Scatterplot showing relationship between H3K4me3 ChIPseq readcounts and mean DNA methylation (WGBS) in 4 kb tiles overlapping H3K4me3-only regions (as defined by Mantsoki et al. in mouse ESC). Linear regression line of best fit is overlayed in blue. Pearson r-squared value is indicated top left. WGBS from WT mouse ESC was generated in-house. (Right) Scatterplot showing relationship between H3K4me3 ChIPseq readcounts and mean DNA methylation (WGBS) in 4 kb tiles overlapping H3K4me3-only regions (as defined by Mantsoki et al. in human ESC). Linear regression line of best fit is overlayed in blue. Pearson r-squared value is indicated top left. Human methylation data was from GEO accession GSM2138820.

Additional file 2: **Figure S2.** Selection of *k* value for *k*-Means clustering. (a) The principle behind *k*-Means clustering is to identify clusters such that the total within-cluster sum of squares (WSS) is minimised. The sum of all WSS relates to cluster compactness and ideally should be as low as possible. Values of *k* are systematically tested until further increases in *k* do not improve the total WSS – the ‘elbow method’. A plot is shown where cluster number (underlying the ChIPseq data in Fig. 2d) is plotted against total WSS with *k* = 2 chosen as optimal for this data. R packages ‘factoextra’ and ‘NBClust’ were implemented for this analysis. (b) To validate the choice of *k* we used the alternative ‘average silhouette method’ approach accounting for cluster quality and inter-cluster distances, which ideally are high and distinct respectively, for robust clustering of non-random data. A plot is shown where cluster number (data underlying Fig. 2d) is plotted against average silhouette width with *k* = 2 returned.

Additional file 3: **Figure S3.** Differential H3K27me3 to H3K4me3 ChIPseq ratios at different genomic resolutions. (a) In Fig. 2d we show differential H3K27me3:H3K4me3 ChIPseq ratios over a genomic window of 4 kb. To test whether this result was due to the differing breadth of H3K27me3 and H3K4me3 ChIPseq peaks we repeated the analysis at 4 kb including two greater resolutions: 0.2 and 1 kb. hiBiv and loBiv were compared using Pearson’s correlation indicating statistically different H3K27me3:H3K4me3 ChIPseq ratios independent of window size. (b) To statistically test if H3K27me3 depletion is linked to transcription start site proximity, we analysed H3K27me3 depletion at three different resolutions. This showed that the greatest difference in H3K27me3 depletion is at the highest genomic distance (+/− 5 kb from peak centre) by boxplot analysis of mean J1:TKO H3K27me3 (rpkm) ratio per locus and non-equal variance two sample Student’s t-Test. Further, largest TKO H3K27me3 alterations occur at the highest DNA methylation level resolution (+/− 5 kb). Over shorter genomic ranges (0.2-1 kb) hiBiv and loBiv differential sensitivity is skewed towards loBiv regions based on median values at lower significance. This is likely due to the marked reduction in Polycomb presence at hiBiv regions, which are relatively dominated by H3K4me3 occupancy over these narrow windows. Mapping of average DNA methylation over the three genomic resolutions indicates that DNA methylation is most abundant over the 10 kb range, implying that DNA methylation is unlikely to play a prominent role in determining Polycomb localisation at the 0.2 and 1 kb ranges.

Additional file 4: **Figure S4.** Quintile chromatin configuration at murine bivalent promoters. As an alternative to *k*-means clustering we divided the data underlying Fig. 2d into H3K27me3:H3K4me3 quintiles determined by the ChIPseq signal ratio. We found that this approach reproduces hiBiv (Q5). As with *k*-means clustering, the Q5 quintile is relatively enriched in PRC components (EZH2, RING1B and uH2A) and depleted of activating marks (H3K4me3 & H2AZ). Q4,Q3,Q2,Q1 account for the loBiv set of regions. KDM2B, which binds to the majority of CGI is relatively uniform across the five quintiles.

Additional file 5: **Table S1.** This additional table contains mouse hiBiv and loBiv genomic coordinates, strand and neighbouring gene.

Additional file 6: **Figure S5.** Overlap between uH2A-retaining loci, mouse hiBiv and mouse loBiv. (a) Genomic region coordinates and associated gene names (*n* = 315) that retain uH2A in mouse ES (mESC) cells lacking PCGF1/3/5/6 were obtained from the Klose lab. Of these, we found *n* = 253 overlapping our *n* = 3659 bivalent genes in mESC (left panel). Specifically, the majority of these (~ 78%) are the hiBiv class, while ~ 22% are the loBiv class – thus regions that uH2A in quadruple PCGF knockout mESC are enriched in the hiBiv class (right panel). (b) Heatmaps for the indicated ChIPseq datasets were plotted without clustering for the regions that retain uH2A in PCGF1/3/5/6 mESC annotated as hiBiv or loBiv. Bottom: average profiles of heatmap data.

Additional file 7: **Figure S6.** Gene ontology analysis of mouse hiBiv and loBiv associated genes. Gorilla and REViGO tools were coupled to define and reduce redundancy for enriched gene ontology terms in murine hiBiv and loBiv compared to all genes. Colour coding of the scatterplots are related to the log10 of the enriched *p*-values estimated by Gorilla. Area of circular plotted data is proportional to the number of genes enriched in each GO term. Redundant GO terms are reduced using the method of ‘semantic similarity’ measurement which is analogous to hierarchical clustering methods. This involves generating clusters of similar GO terms based on close p-values and whether one term is a child node of the other term [76].

Additional file 8: **Figure S7.** Dynamics of bivalency during early embryonic development. Related to Fig. 4a. A similar approach adopted in Fig. 2d was used to compute H3K27me3:H3K4me3 ratio based on mean RPKM values in hiBiv and loBiv loci. Reciprocal ratios of H3K4me3:H3K27me3 were also computed. Upper panel: hiBiv, lower panel: loBiv.

Additional file 9: **Figure S8.** Human ESC hiBiv and loBiv defined regions. (a) Human H3K27me3 and H3K4me3 ChIPseq datasets derived from H1 and H9 human ESCs described in *Mantsoki* et al. [45]. were downloaded and mapped using bowtie2 [45]. Multiple mapped files (bam files) from each histone modification were merged and indexed using Samtools. Coverage files (bigWig) and coverage matrices were computed with deepTools using the human bivalent regions described in *Mantsoki* et al [45]. (b) Human H3K27me3 and H3K4me3 ChIPseq datasets derived from H1 human ESCs were downloaded from ENCODE (Bing Ren, UCSD) and mapped using bowtie2. Multiple mapped files (bam files) from each histone modification were merged and indexed using Samtools. Coverage files (bigWig) and coverage matrices were computed with deepTools using the human bivalent regions described in *Mantsoki* et al. [45].

Additional file 10: **Table S2.** This additional table contains: human hiBiv and loBiv genomic coordinates, strand and neighbouring gene.

Additional file 11: **Figure S9.** Gene ontology analysis of human hiBiv and loBiv associated genes. Gorilla and REViGO tools were coupled to define and reduce redundancy for enriched gene ontology terms in human hiBiv and loBiv compared to all genes. Colour coding of the scatterplots are related to the log10 of the enriched p-values estimated by Gorilla. Area of circular plotted data is proportional to the number of genes enriched in each GO term. Redundant GO terms are reduced using the method of ‘semantic similarity’ measurement which is analogous to hierarchical clustering methods. This involves generating clusters of similar GO terms based on close p-values and whether one term is a child node of the other term [76].

Additional file 12: **Figure S10.** Quintile chromatin configuration at human bivalent promoters. Left panel: As an alternative to *k*-means clustering we divided the data underlying Fig. S8 into H3K27me3:H3K4me3 quintiles determined by the ChIPseq signal ratio which emphasises the difference between Q5 and Q4 to Q1 inclusive. Q5 is consistent with hiBiv and Q4 to Q1 account for loBiv. Right panel: we performed a similar quintile division of the data underlying Fig. 6a. Q5 quintile (which is most related to hiBiv) shows most H3K27me3-sensitivity to DNA hypomethylation comparing HMEC to HCC1954/MCF7 and colon to HCT116. In contrast, Q4 to Q1 show an attenuated H3K27me3-response to DNA hypomethylation.

Additional file 13: **Figure S11.** Reproduction of TCGA breast methylation data from Fig. 6f including high density dendograms. Shown are the breast analyses from Fig. 6f including dendograms which indicate clustered loci (y-axis) and controls/patients (x-axis).

Additional file 14: **Figure S12.** Reproduction of TCGA colon methylation data from Fig. 6f including high density dendograms. Shown are the colon analyses from Fig. 6f including dendograms which indicate clustered loci (y-axis) and controls/patients (x-axis).

Additional file 15: **Figure S13.** Absence of hypermethylation at H4Kme3-only and H3K27me3-only promoters. To test if cancer-specific DNA hypermethylation was more associated with high ratio H3K27me3:H3K4me3 loci (hiBiv) than singly marked promoters, we analysed promoters characterised by the enrichment of either K4me3-only or H3K27me3-only in human ES cells. We repeated our approach detailed in Fig. 6f and found little evidence of promoter hypermethylation in either breast or colon tumours at these loci.

Additional file 16: **Figure S14.** hiBiv subclass of bivalent promoters is most susceptible to hypermethylation in cancer. We analysed degree of hypermethylation and hypomethylation (as defined by a 20% or greater alteration in mean DNA methylation per locus) in hiBiv, loBiv, H3K27me3 only, H3K4me3 only and latent (neither H3K27me3 nor H3K4me3) loci (as defined by their demarcation in human ES cells). We next computed the percentage of loci within each promoter class altered. Promoters with greatest cancer hypermethylation events occur in the hiBiv class (27% of all colon hiBiv loci; 8% of all breast hiBiv loci) followed by the loBiv class (13% of all colon hiBiv loci; 2% of all breast hiBiv loci). Interestingly, H3K27 only and latent loci exhibited the highest degree of DNA hypomethylation events.

Additional file 17: **Table S3.** This additional table contains datasets re-analysed in this manuscript. File format.
