# Supplementary figures and images for: Bivalent promoter hypermethylation in cancer is linked to the H327me3/H3K4me3 ratio in embryonic stem cells
Source: BMC Biol. 2020 Mar 4;18:25. doi: 10.1186/s12915-020-0752-3 (PMC7057567; doi:10.1186/s12915-020-0752-3)

Plot of WT mouse ESC H3K4me3 versus 5meC

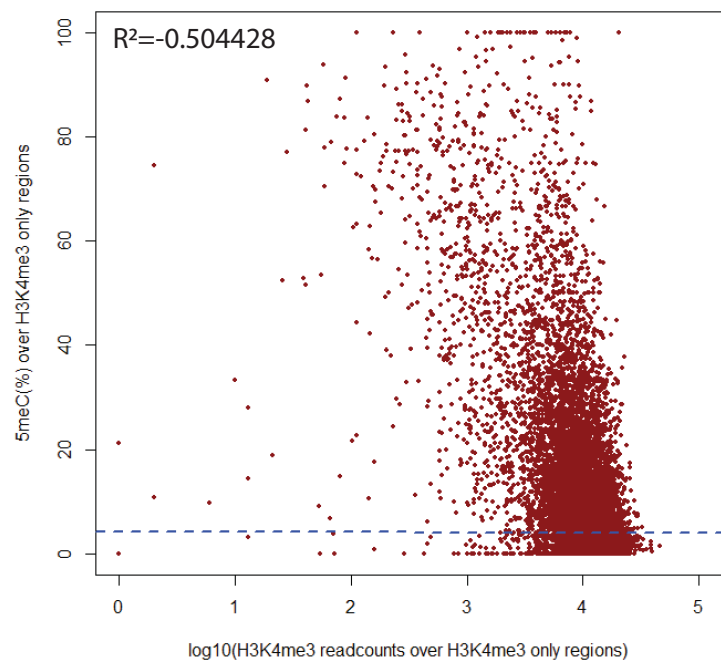

Plot of WT human ESC H3K4me3 versus 5meC

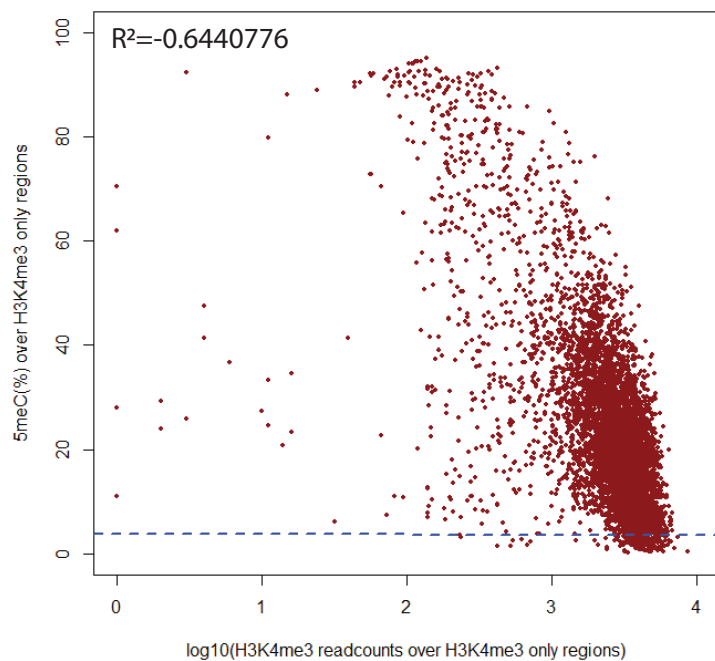

Supplement: Supplementary file 1 — Figure S1. Negative correlation between H3K4me3-only enriched regions and DNA methylation in mouse and human ESC. (Left) Scatterplot showing relationship between H3K4me3 ChIPseq readcounts and mean DNA methylation (WGBS) in 4 kb tiles overlapping H3K4me3-only regions (as defined by Mantsoki et al. in mouse ESC). Linear regression line of best fit is overlayed in blue. Pearson r-squared value is indicated top left. WGBS from WT mouse ESC was generated in-house. (Right) Scatterplot showing relationship between H3K4me3 ChIPseq readcounts and mean DNA methylation (WGBS) in 4 kb tiles overlapping H3K4me3-only regions (as defined by Mantsoki et al. in human ESC). Linear regression line of best fit is overlayed in blue. Pearson r-squared value is indicated top left. Human methylation data was from GEO accession GSM2138820. [file 12915_2020_752_MOESM1_ESM.pdf]

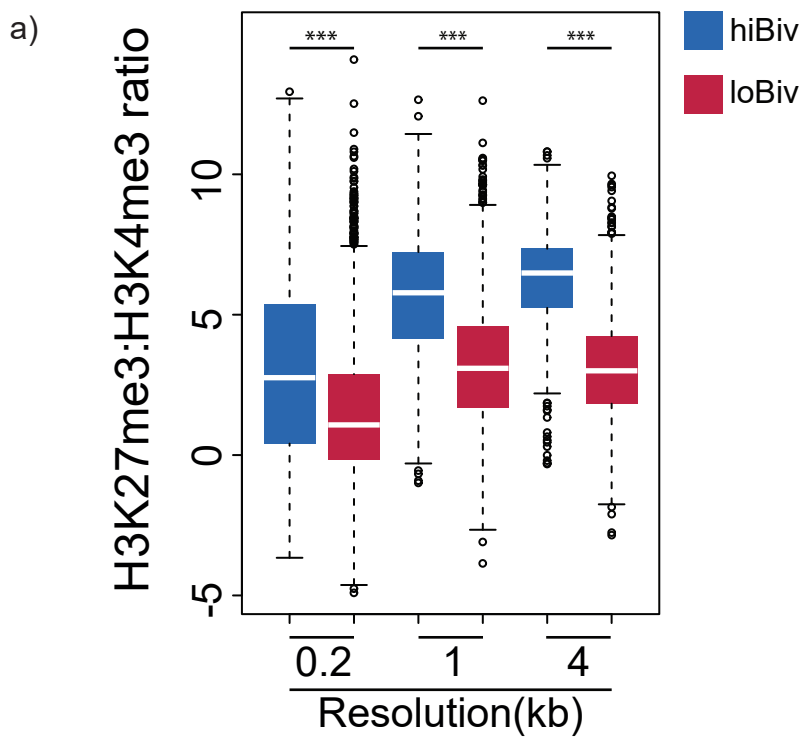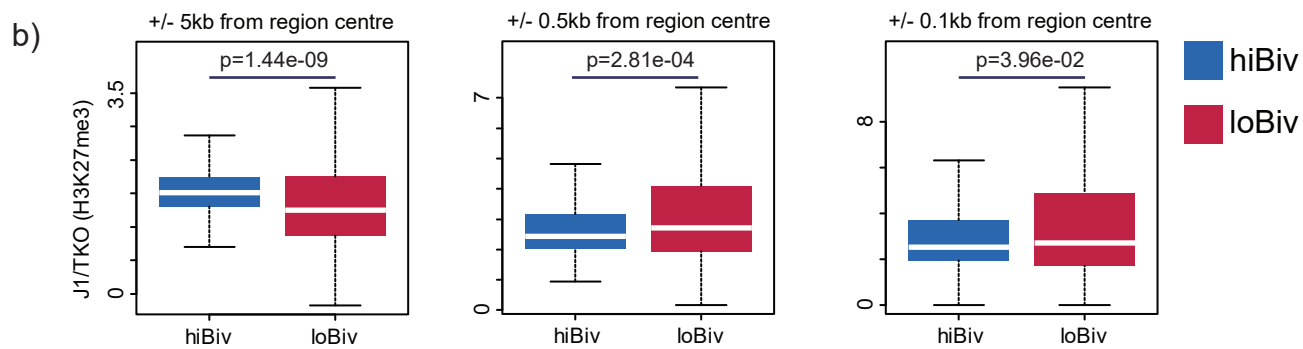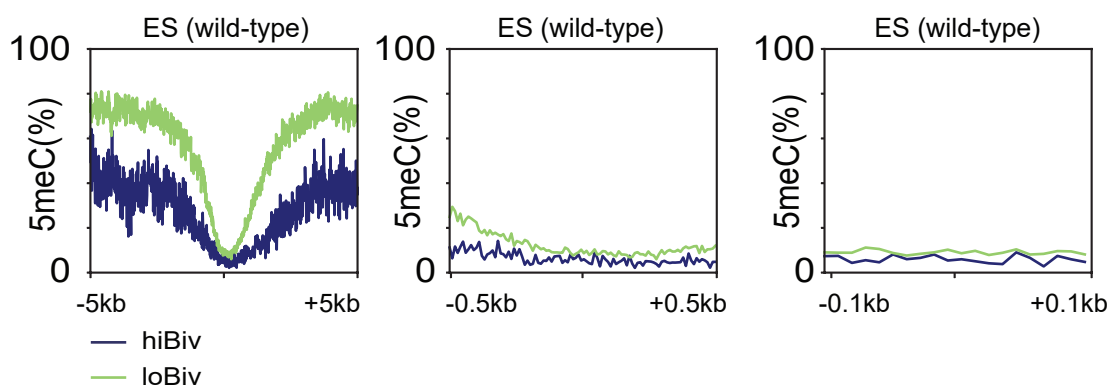

Supplement: Supplementary file 3 — Figure S3. Differential H3K27me3 to H3K4me3 ChIPseq ratios at different genomic resolutions. (a) In Fig. 2d we show differential H3K27me3:H3K4me3 ChIPseq ratios over a genomic window of 4 kb. To test whether this result was due to the differing breadth of H3K27me3 and H3K4me3 ChIPseq peaks we repeated the analysis at 4 kb including two greater resolutions: 0.2 and 1 kb. hiBiv and loBiv were compared using Pearson’s correlation indicating statistically different H3K27me3:H3K4me3 ChIPseq ratios independent of window size. (b) To statistically test if H3K27me3 depletion is linked to transcription start site proximity, we analysed H3K27me3 depletion at three different resolutions. This showed that the greatest difference in H3K27me3 depletion is at the highest genomic distance (+/− 5 kb from peak centre) by boxplot analysis of mean J1:TKO H3K27me3 (rpkm) ratio per locus and non-equal variance two sample Student’s t-Test. Further, largest TKO H3K27me3 alterations occur at the highest DNA methylation level resolution (+/− 5 kb). Over shorter genomic ranges (0.2-1 kb) hiBiv and loBiv differential sensitivity is skewed towards loBiv regions based on median values at lower significance. This is likely due to the marked reduction in Polycomb presence at hiBiv regions, which are relatively dominated by H3K4me3 occupancy over these narrow windows. Mapping of average DNA methylation over the three genomic resolutions indicates that DNA methylation is most abundant over the 10 kb range, implying that DNA methylation is unlikely to play a prominent role in determining Polycomb localisation at the 0.2 and 1 kb ranges. [file 12915_2020_752_MOESM3_ESM.pdf]

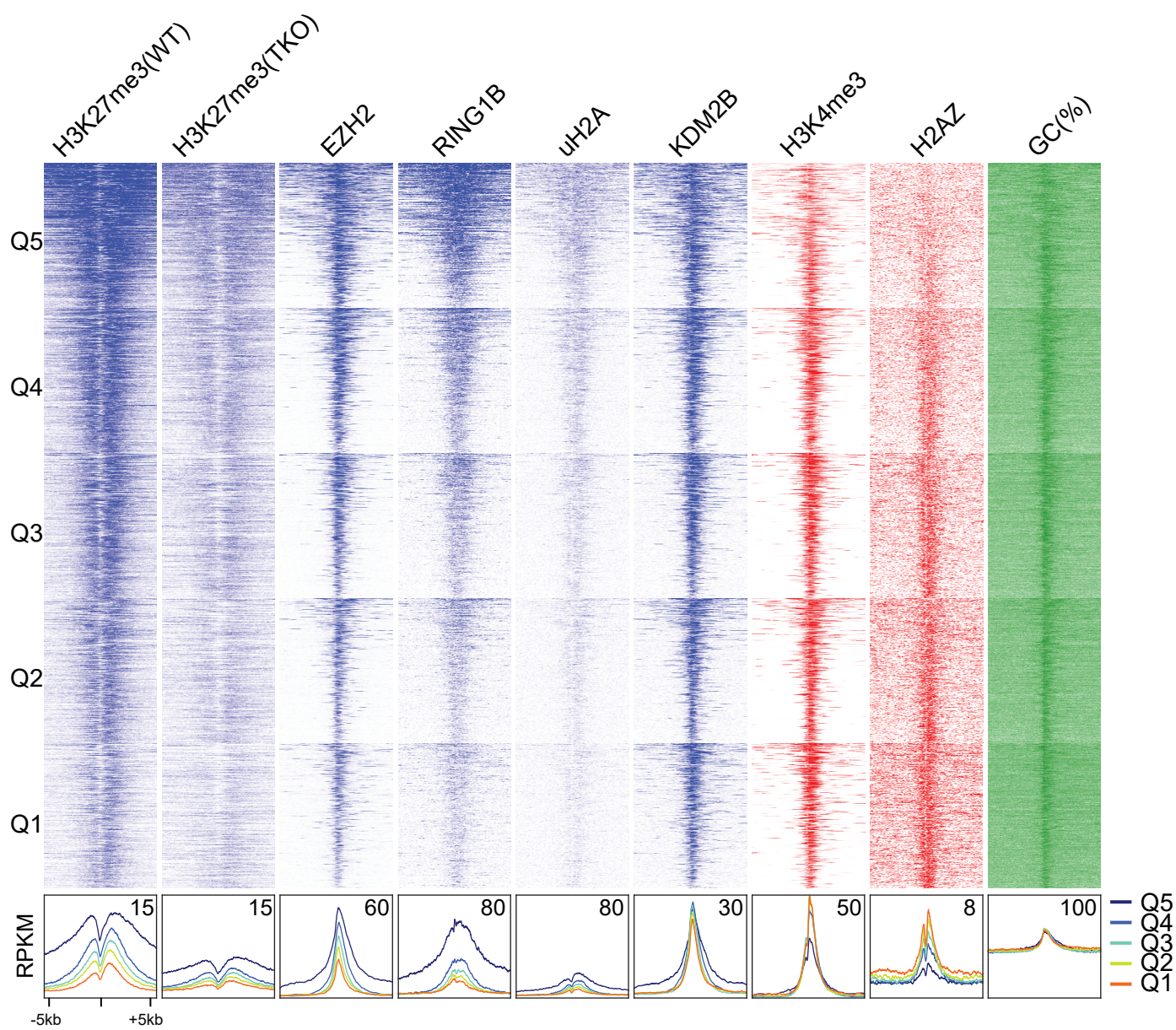

Supplement: Supplementary file 4 — Figure S4. Quintile chromatin configuration at murine bivalent promoters. As an alternative to k-means clustering we divided the data underlying Fig. 2d into H3K27me3:H3K4me3 quintiles determined by the ChIPseq signal ratio. We found that this approach reproduces hiBiv (Q5). As with k-means clustering, the Q5 quintile is relatively enriched in PRC components (EZH2, RING1B and uH2A) and depleted of activating marks (H3K4me3 & H2AZ). Q4,Q3,Q2,Q1 account for the loBiv set of regions. KDM2B, which binds to the majority of CGI is relatively uniform across the five quintiles. [file 12915_2020_752_MOESM4_ESM.pdf]

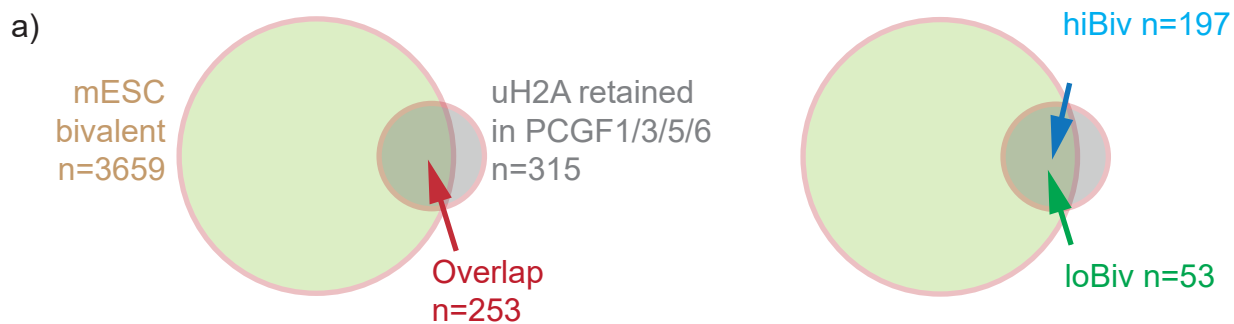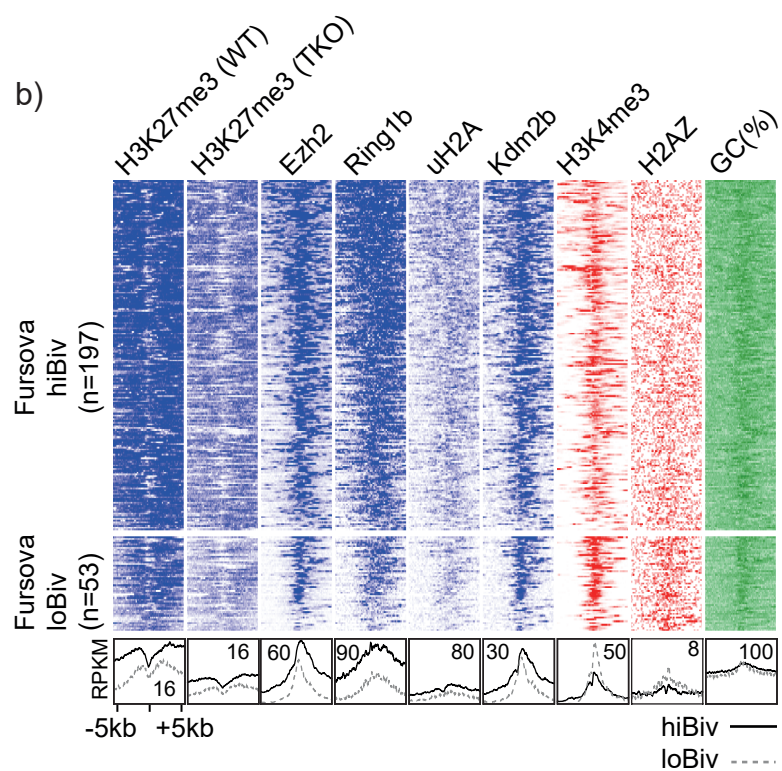

Supplement: Supplementary file 6 — Figure S5. Overlap between uH2A-retaining loci, mouse hiBiv and mouse loBiv. (a) Genomic region coordinates and associated gene names (n = 315) that retain uH2A in mouse ES (mESC) cells lacking PCGF1/3/5/6 were obtained from the Klose lab. Of these, we found n = 253 overlapping our n = 3659 bivalent genes in mESC (left panel). Specifically, the majority of these (~ 78%) are the hiBiv class, while ~ 22% are the loBiv class – thus regions that uH2A in quadruple PCGF knockout mESC are enriched in the hiBiv class (right panel). (b) Heatmaps for the indicated ChIPseq datasets were plotted without clustering for the regions that retain uH2A in PCGF1/3/5/6 mESC annotated as hiBiv or loBiv. Bottom: average profiles of heatmap data. [file 12915_2020_752_MOESM6_ESM.pdf]

Mouse hiBiv

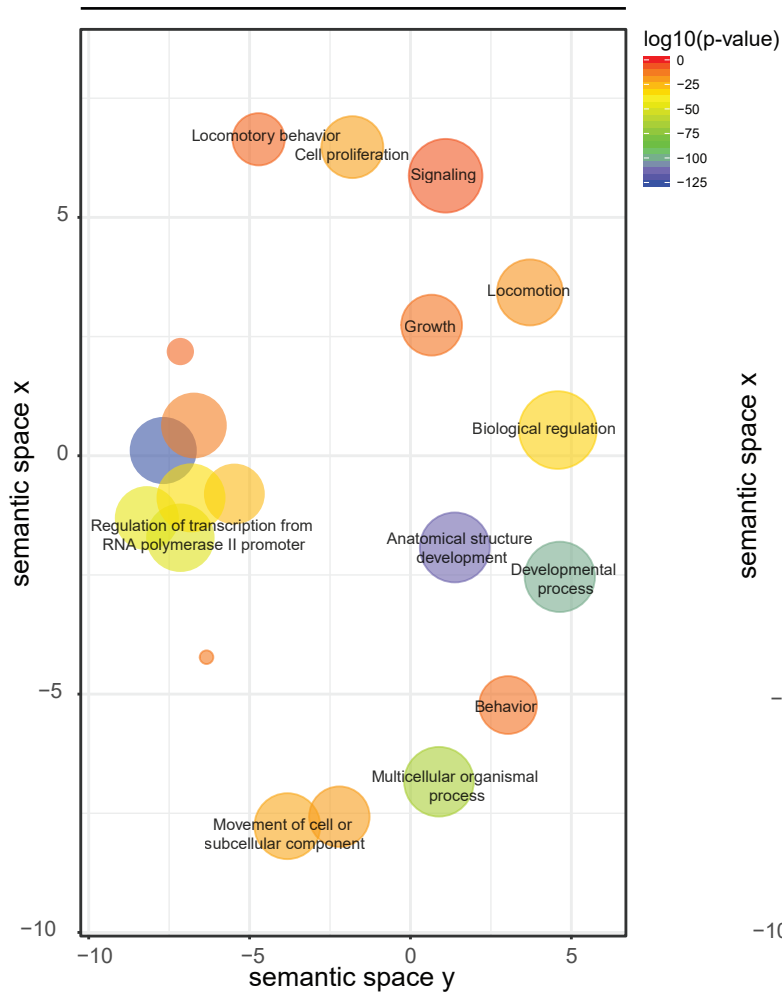

Mouse loBiv

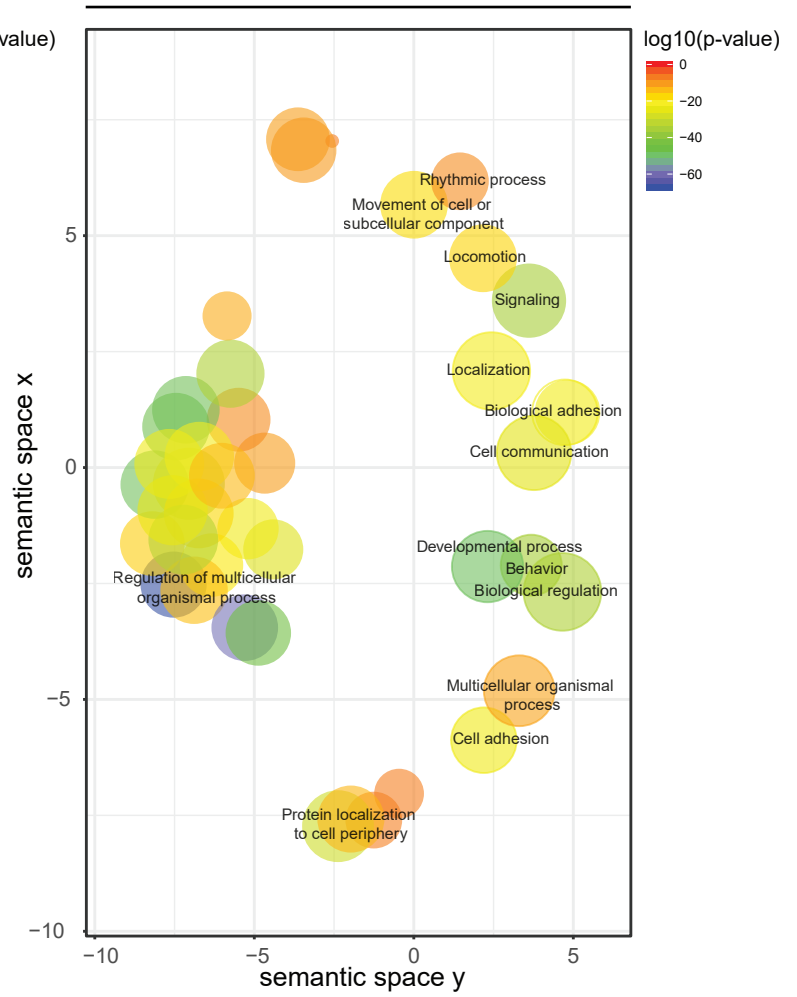

Supplement: Supplementary file 7 — Figure S6. Gene ontology analysis of mouse hiBiv and loBiv associated genes. Gorilla and REViGO tools were coupled to define and reduce redundancy for enriched gene ontology terms in murine hiBiv and loBiv compared to all genes. Colour coding of the scatterplots are related to the log10 of the enriched p-values estimated by Gorilla. Area of circular plotted data is proportional to the number of genes enriched in each GO term. Redundant GO terms are reduced using the method of ‘semantic similarity’ measurement which is analogous to hierarchical clustering methods. This involves generating clusters of similar GO terms based on close p-values and whether one term is a child node of the other term [76]. [file 12915_2020_752_MOESM7_ESM.pdf]

hiBiv

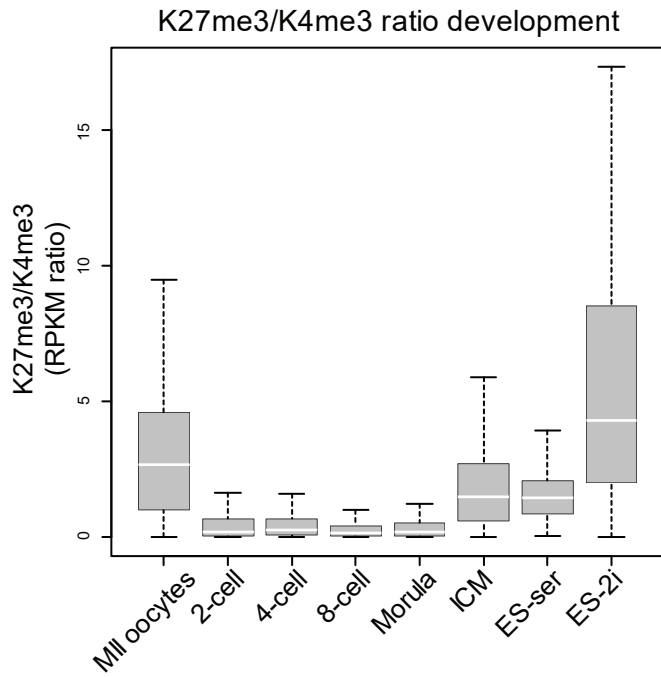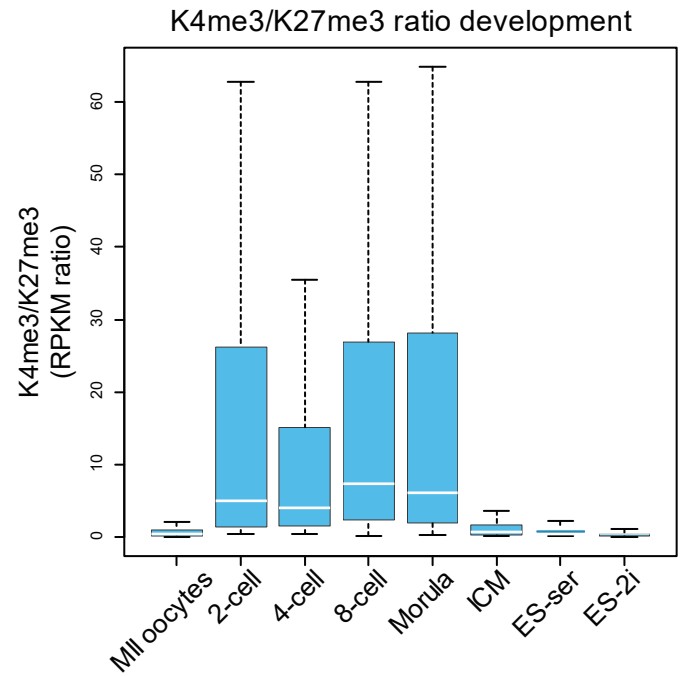

loBiv

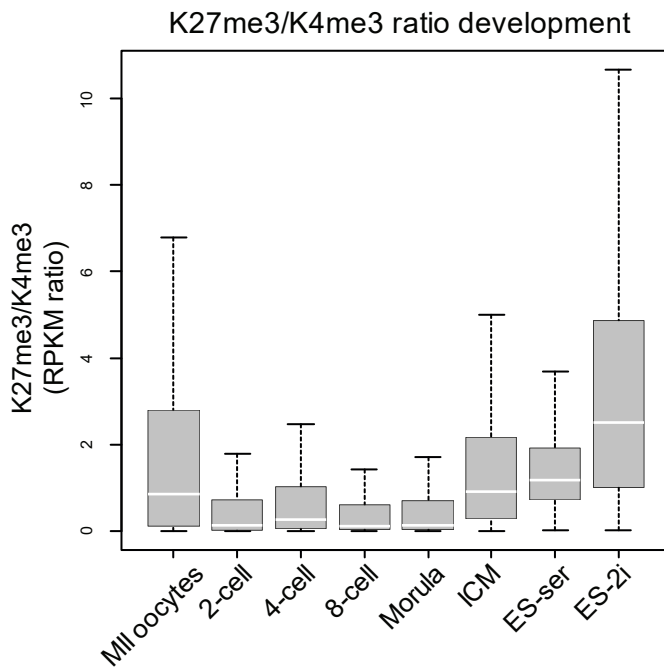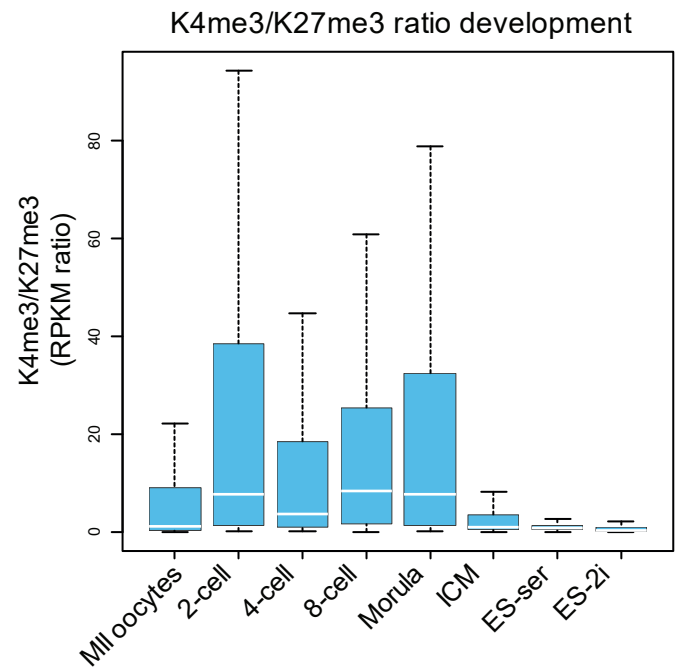

Supplement: Supplementary file 8 — Figure S7. Dynamics of bivalency during early embryonic development. Related to Fig. 4a. A similar approach adopted in Fig. 2d was used to compute H3K27me3:H3K4me3 ratio based on mean RPKM values in hiBiv and loBiv loci. Reciprocal ratios of H3K4me3:H3K27me3 were also computed. Upper panel: hiBiv, lower panel: loBiv. [file 12915_2020_752_MOESM8_ESM.pdf]

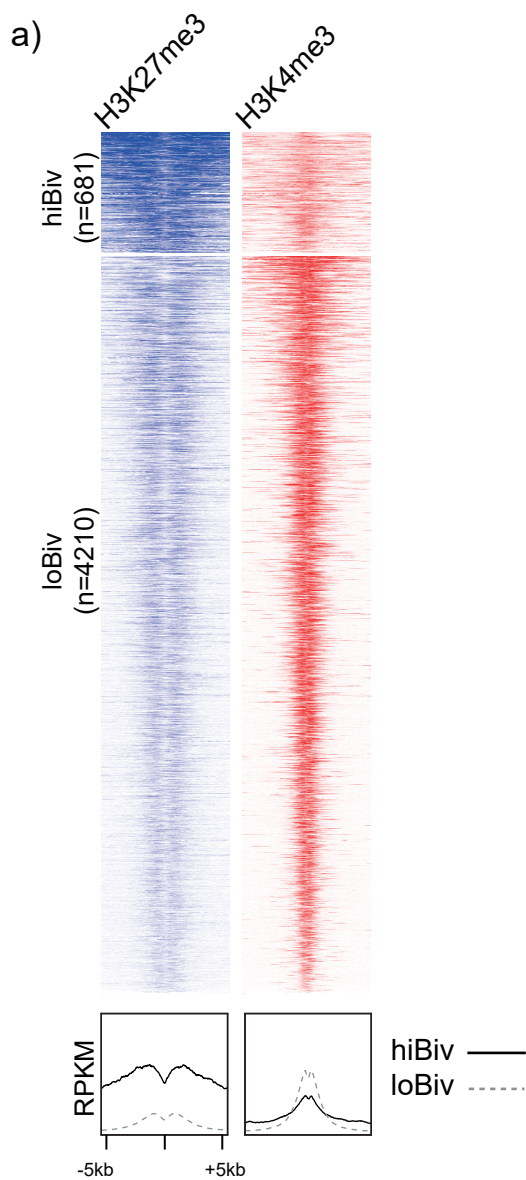

Mantsoki et al. H1 & H9 hESC

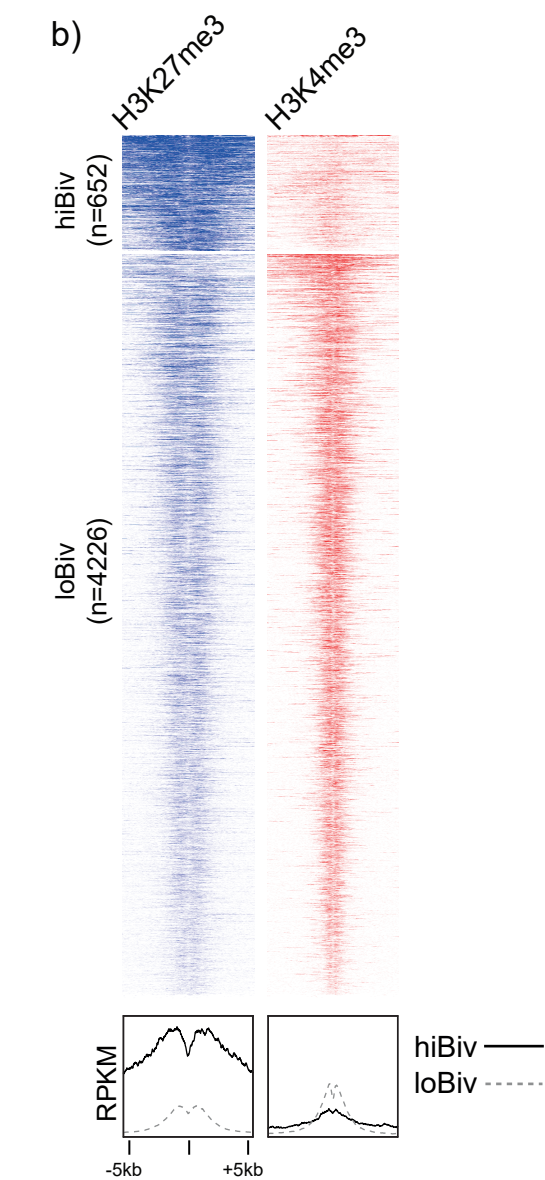

ENCODE (Bing Ren) H1 hESC

Supplement: Supplementary file 9 — Figure S8. Human ESC hiBiv and loBiv defined regions. (a) Human H3K27me3 and H3K4me3 ChIPseq datasets derived from H1 and H9 human ESCs described in Mantsoki et al. [45]. were downloaded and mapped using bowtie2 [45]. Multiple mapped files (bam files) from each histone modification were merged and indexed using Samtools. Coverage files (bigWig) and coverage matrices were computed with deepTools using the human bivalent regions described in Mantsoki et al [45]. (b) Human H3K27me3 and H3K4me3 ChIPseq datasets derived from H1 human ESCs were downloaded from ENCODE (Bing Ren, UCSD) and mapped using bowtie2. Multiple mapped files (bam files) from each histone modification were merged and indexed using Samtools. Coverage files (bigWig) and coverage matrices were computed with deepTools using the human bivalent regions described in Mantsoki et al. [45]. [file 12915_2020_752_MOESM9_ESM.pdf]

Human hiBiv

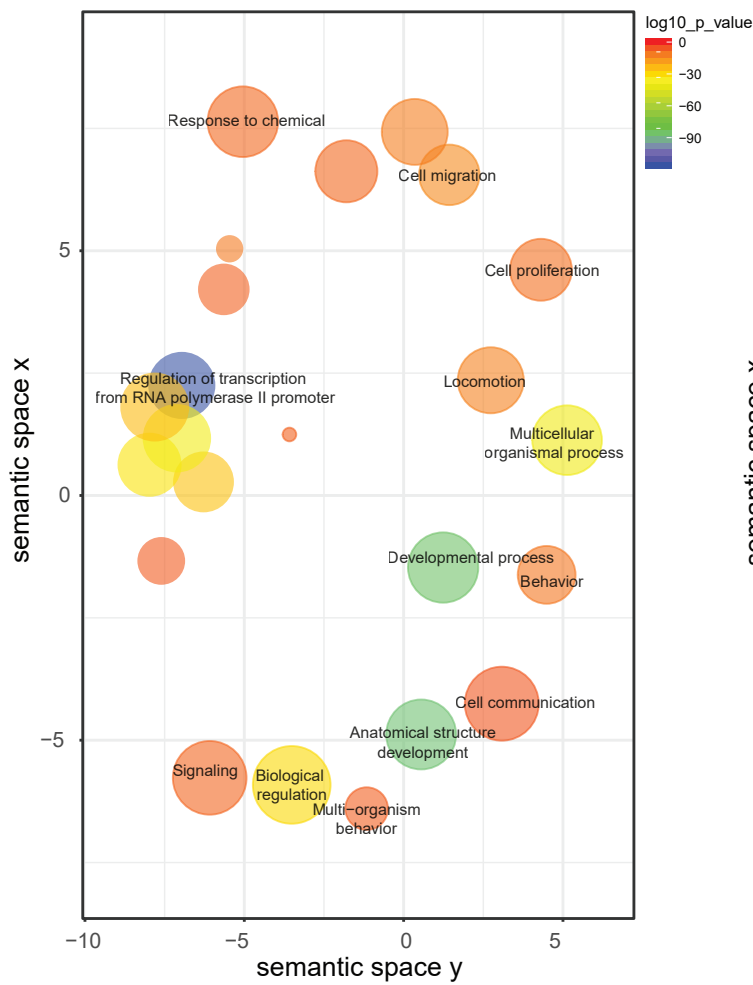

Human loBiv

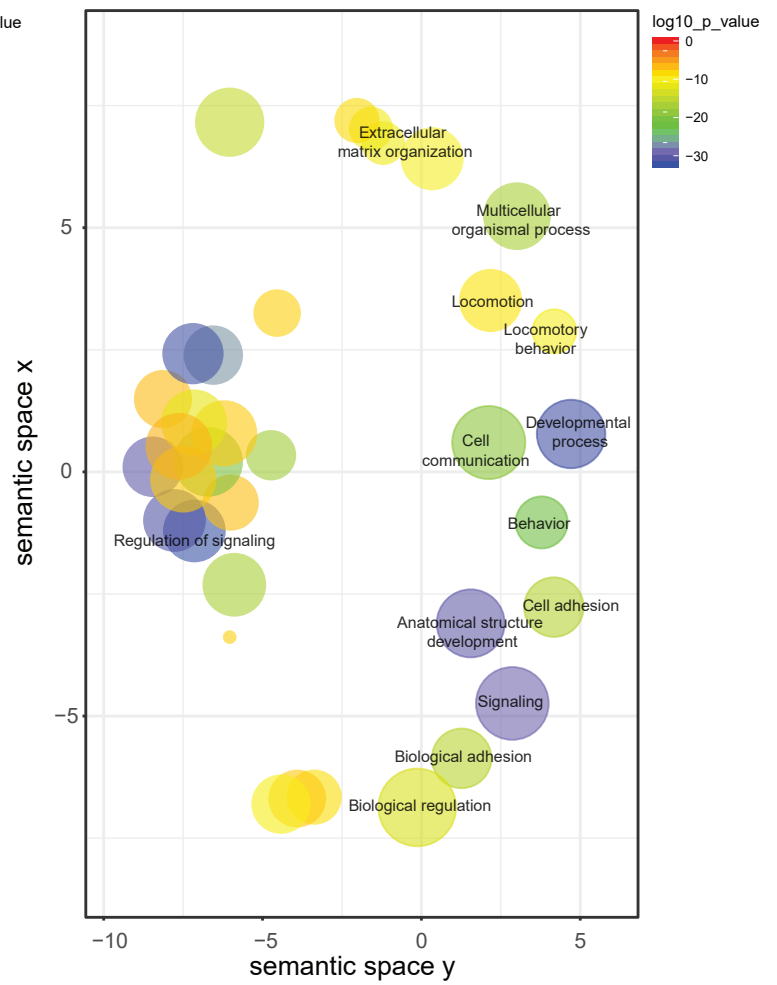

Supplement: Supplementary file 11 — Figure S9. Gene ontology analysis of human hiBiv and loBiv associated genes. Gorilla and REViGO tools were coupled to define and reduce redundancy for enriched gene ontology terms in human hiBiv and loBiv compared to all genes. Colour coding of the scatterplots are related to the log10 of the enriched p-values estimated by Gorilla. Area of circular plotted data is proportional to the number of genes enriched in each GO term. Redundant GO terms are reduced using the method of ‘semantic similarity’ measurement which is analogous to hierarchical clustering methods. This involves generating clusters of similar GO terms based on close p-values and whether one term is a child node of the other term [76]. [file 12915_2020_752_MOESM11_ESM.pdf]

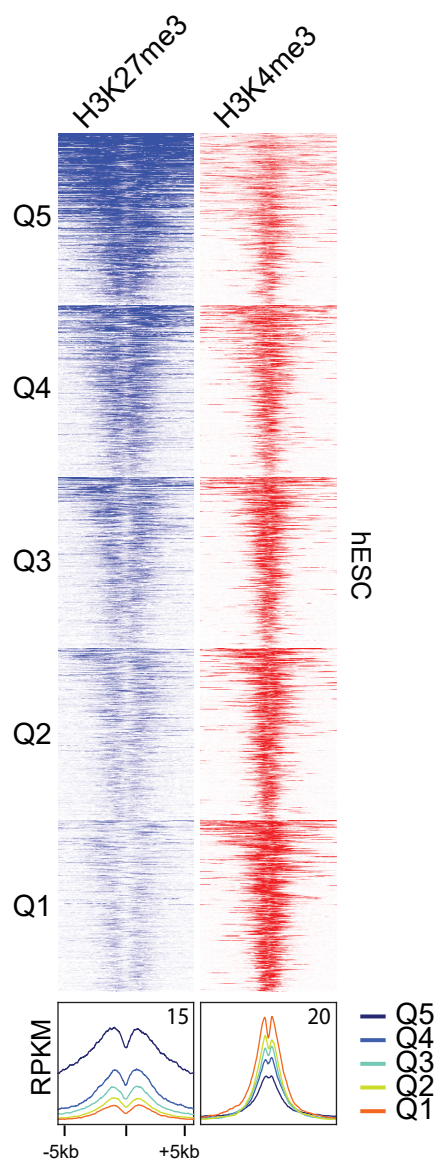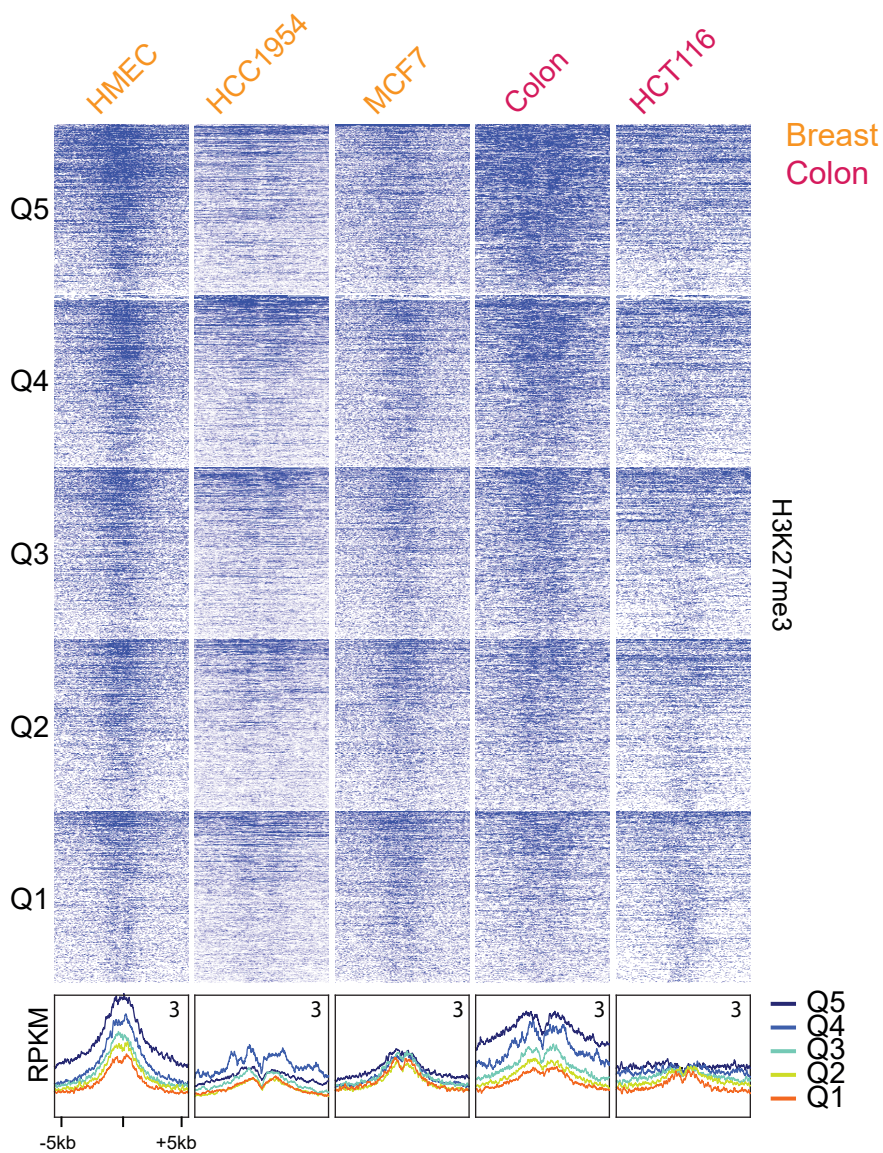

Supplement: Supplementary file 12 — Figure S10. Quintile chromatin configuration at human bivalent promoters. Left panel: As an alternative to k-means clustering we divided the data underlying Fig. S8 into H3K27me3:H3K4me3 quintiles determined by the ChIPseq signal ratio which emphasises the difference between Q5 and Q4 to Q1 inclusive. Q5 is consistent with hiBiv and Q4 to Q1 account for loBiv. Right panel: we performed a similar quintile division of the data underlying Fig. 6a. Q5 quintile (which is most related to hiBiv) shows most H3K27me3-sensitivity to DNA hypomethylation comparing HMEC to HCC1954/MCF7 and colon to HCT116. In contrast, Q4 to Q1 show an attenuated H3K27me3-response to DNA hypomethylation. [file 12915_2020_752_MOESM12_ESM.pdf]

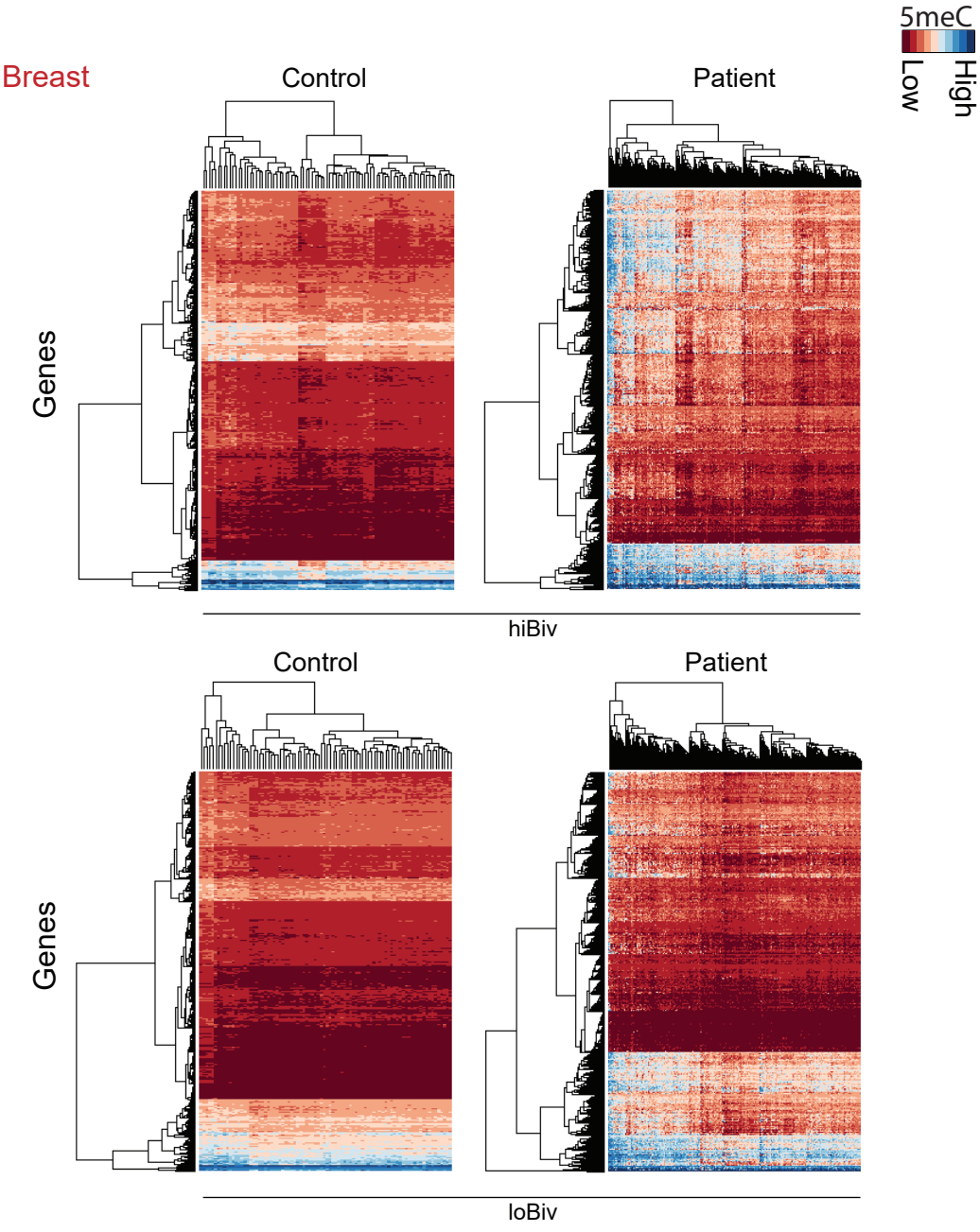

Supplement: Supplementary file 13 — Figure S11. Reproduction of TCGA breast methylation data from Fig. 6f including high density dendograms. Shown are the breast analyses from Fig. 6f including dendograms which indicate clustered loci (y-axis) and controls/patients (x-axis). [file 12915_2020_752_MOESM13_ESM.pdf]

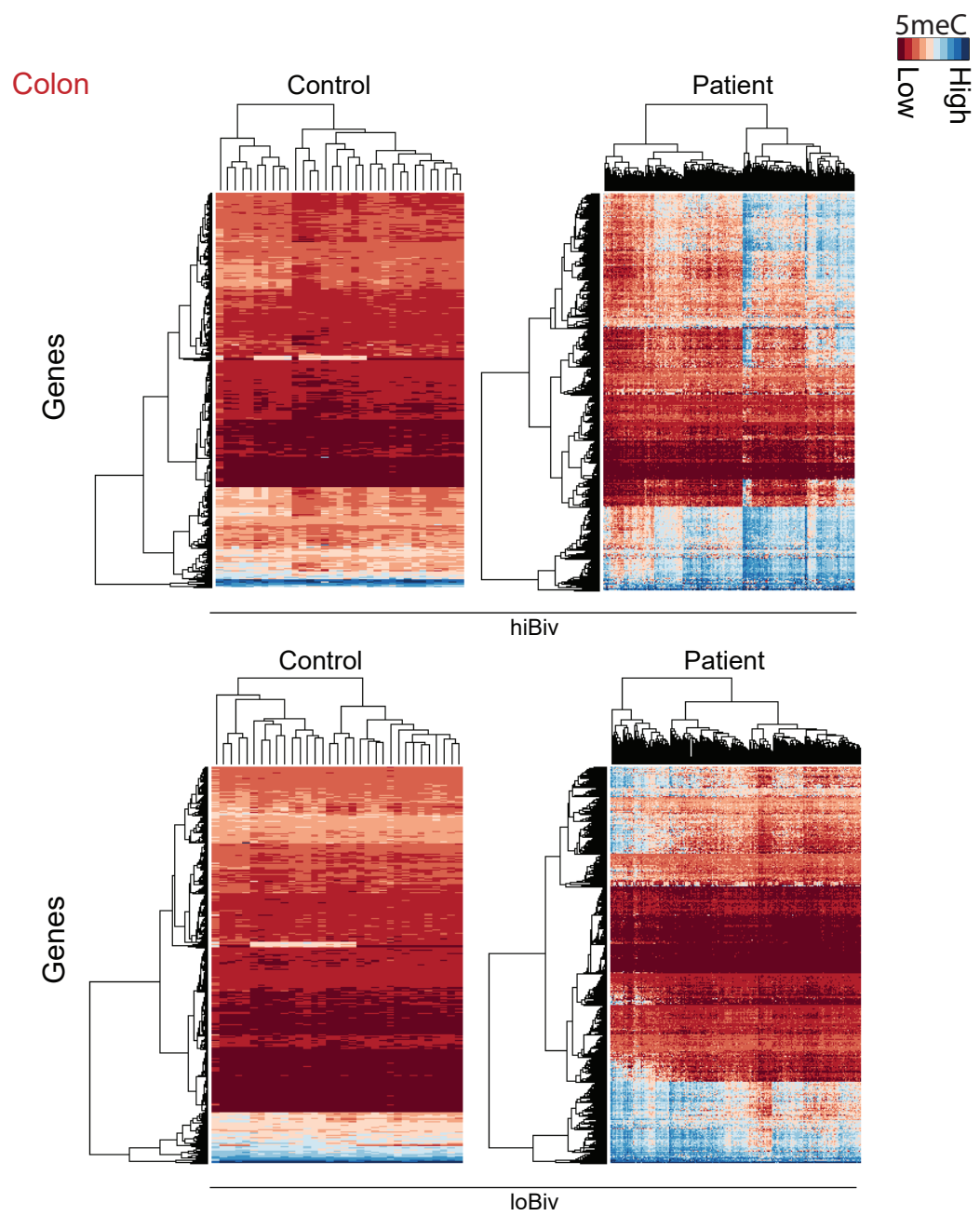

Supplement: Supplementary file 14 — Figure S12. Reproduction of TCGA colon methylation data from Fig. 6f including high density dendograms. Shown are the colon analyses from Fig. 6f including dendograms which indicate clustered loci (y-axis) and controls/patients (x-axis). [file 12915_2020_752_MOESM14_ESM.pdf]

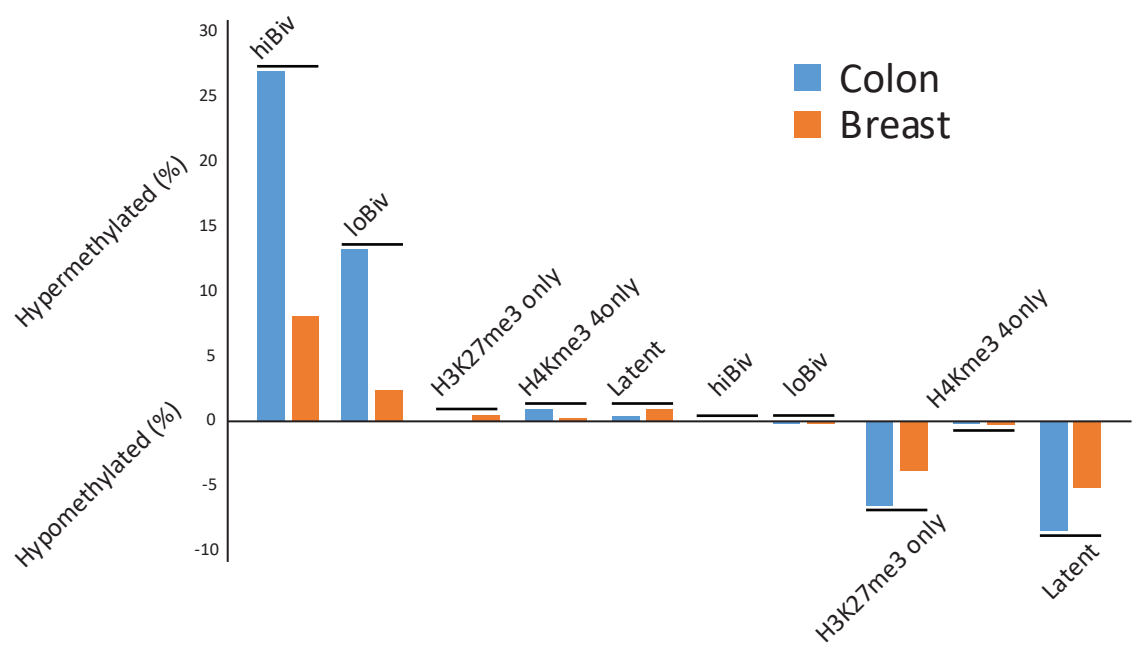

Supplement: Supplementary file 16 — Figure S14. hiBiv subclass of bivalent promoters is most susceptible to hypermethylation in cancer. We analysed degree of hypermethylation and hypomethylation (as defined by a 20% or greater alteration in mean DNA methylation per locus) in hiBiv, loBiv, H3K27me3 only, H3K4me3 only and latent (neither H3K27me3 nor H3K4me3) loci (as defined by their demarcation in human ES cells). We next computed the percentage of loci within each promoter class altered. Promoters with greatest cancer hypermethylation events occur in the hiBiv class (27% of all colon hiBiv loci; 8% of all breast hiBiv loci) followed by the loBiv class (13% of all colon hiBiv loci; 2% of all breast hiBiv loci). Interestingly, H3K27 only and latent loci exhibited the highest degree of DNA hypomethylation events. [file 12915_2020_752_MOESM16_ESM.pdf]
