# Supplementary material for: Bivalent promoter hypermethylation in cancer is linked to the H327me3/H3K4me3 ratio in embryonic stem cells
Source: BMC Biol. 2020 Mar 4;18:25. doi: 10.1186/s12915-020-0752-3 (PMC7057567; doi:10.1186/s12915-020-0752-3)
Supplement: Supplementary file 2 — Figure S2. Selection of k value for k-Means clustering. (a) The principle behind k-Means clustering is to identify clusters such that the total within-cluster sum of squares (WSS) is minimised. The sum of all WSS relates to cluster compactness and ideally should be as low as possible. Values of k are systematically tested until further increases in k do not improve the total WSS – the ‘elbow method’. A plot is shown where cluster number (underlying the ChIPseq data in Fig. 2d) is plotted against total WSS with k = 2 chosen as optimal for this data. R packages ‘factoextra’ and ‘NBClust’ were implemented for this analysis. (b) To validate the choice of k we used the alternative ‘average silhouette method’ approach accounting for cluster quality and inter-cluster distances, which ideally are high and distinct respectively, for robust clustering of non-random data. A plot is shown where cluster number (data underlying Fig. 2d) is plotted against average silhouette width with k = 2 returned. [file 12915_2020_752_MOESM2_ESM.pdf]

a)

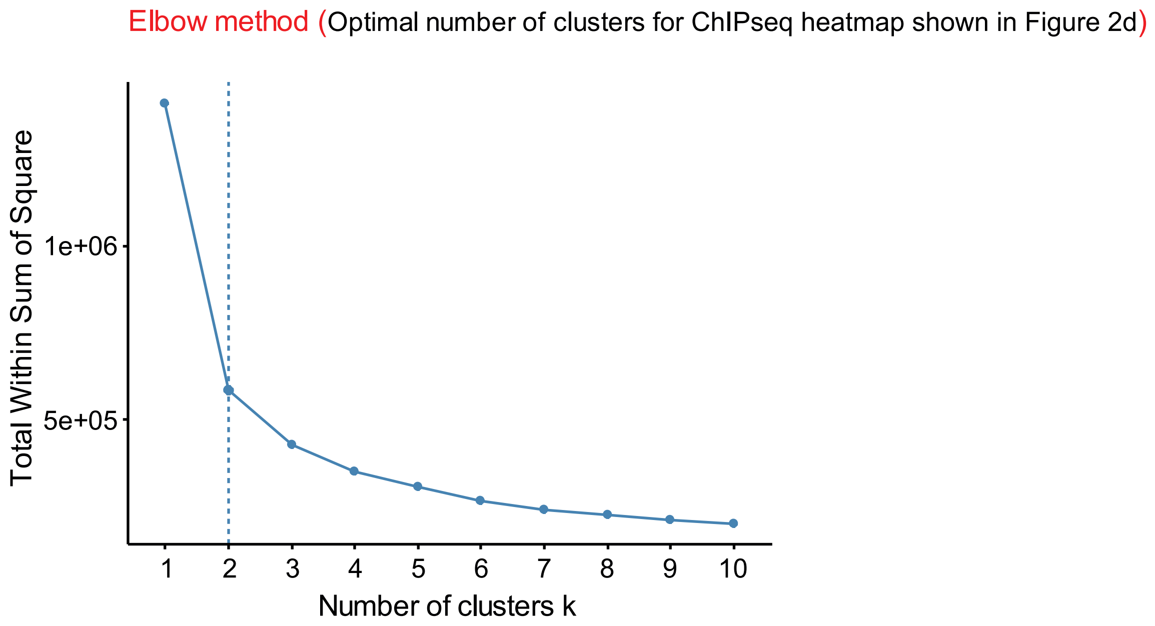

Elbow method (Optimal number of clusters for ChIPseq heatmap shown in Figure 2d)
Total Within Sum of Square
1e+06
5e+05
1
2
3
4
5
6
7
8
9
10
Number of clusters k

b)

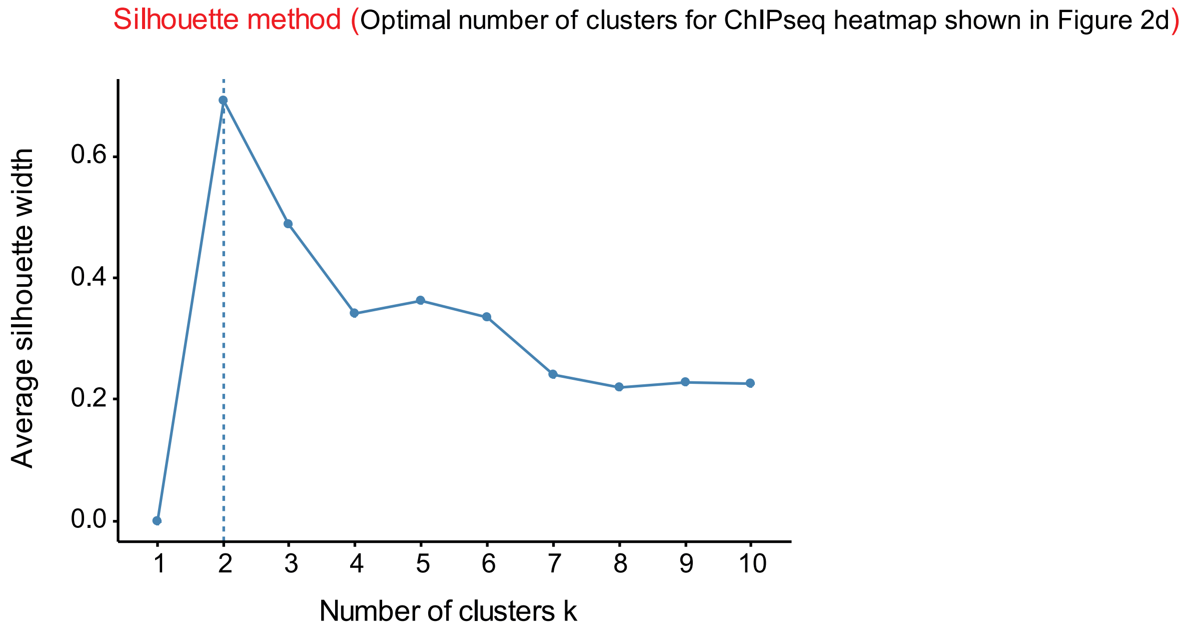

Silhouette method (Optimal number of clusters for ChIPseq heatmap shown in Figure 2d)
Average silhouette width
0.6
0.4
0.2
0.0
1
2
3
4
5
6
7
8
9
10
Number of clusters k
