# Supplementary material for: Bivalent promoter hypermethylation in cancer is linked to the H327me3/H3K4me3 ratio in embryonic stem cells
Source: BMC Biol. 2020 Mar 4;18:25. doi: 10.1186/s12915-020-0752-3 (PMC7057567; doi:10.1186/s12915-020-0752-3)
Supplement: Supplementary file 15 — Figure S13. Absence of hypermethylation at H4Kme3-only and H3K27me3-only promoters. To test if cancer-specific DNA hypermethylation was more associated with high ratio H3K27me3:H3K4me3 loci (hiBiv) than singly marked promoters, we analysed promoters characterised by the enrichment of either K4me3-only or H3K27me3-only in human ES cells. We repeated our approach detailed in Fig. 6f and found little evidence of promoter hypermethylation in either breast or colon tumours at these loci. [file 12915_2020_752_MOESM15_ESM.pdf]

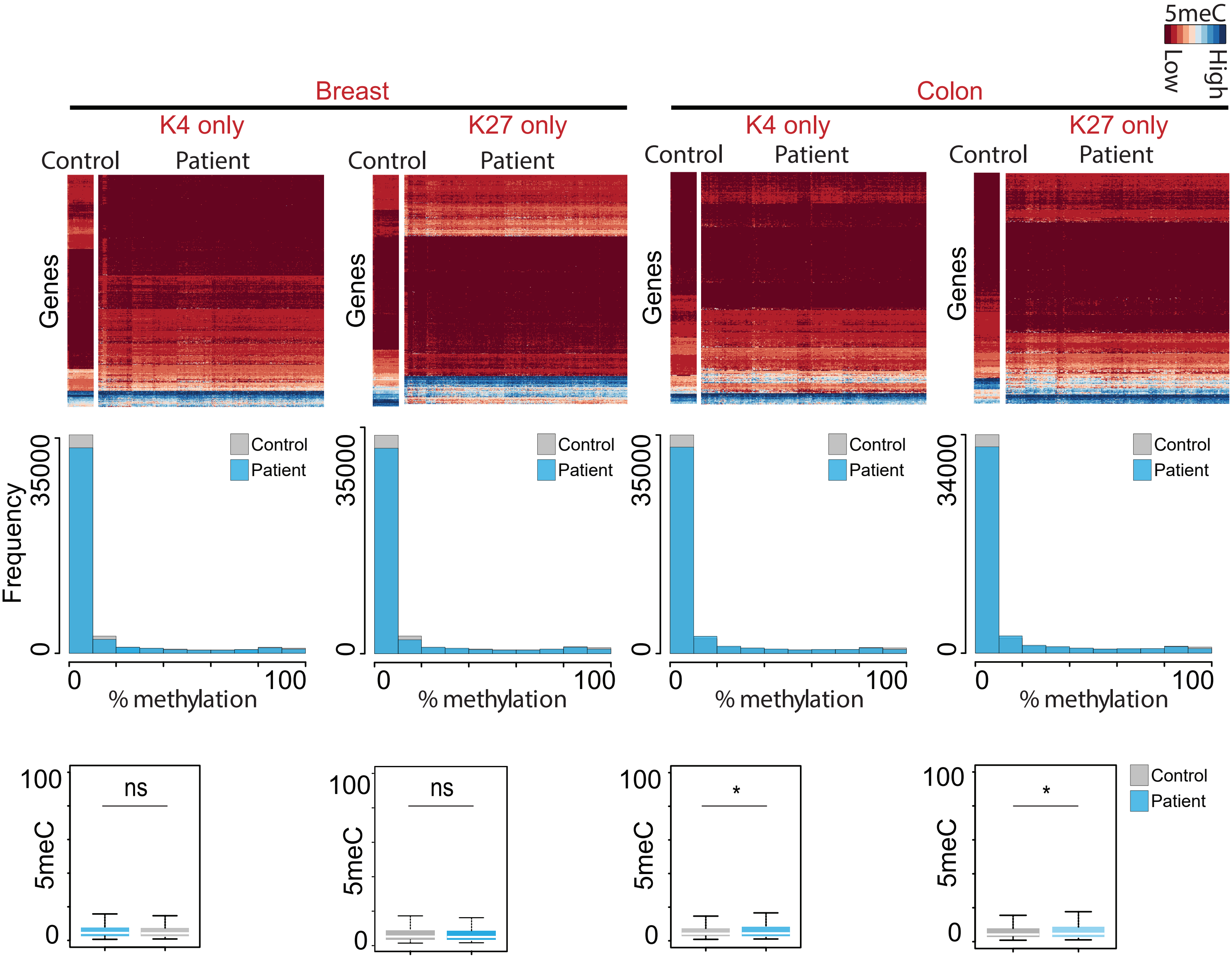
5meC
Low
High
Breast
Colon
K4 only
K27 only
K4 only
K27 only
Control
Patient
Control
Patient
Control
Patient
Control
Patient
Genes
Genes
Genes
Genes
Frequency
35000
0
35000
0
35000
0
34000
0
Control
Patient
Control
Patient
Control
Patient
Control
Patient
0
100
% methylation
0
100
% methylation
0
100
% methylation
0
100
% methylation
100
5meC
0
ns
100
5meC
0
ns
100
5meC
0
*
100
5meC
0
*
Control
Patient
